# Supplementary figures and images for: Distribution of vitamin D status in the UK: a cross-sectional analysis of UK Biobank
Source: BMJ Open. 2021 Jan 6;11(1):e038503. doi: 10.1136/bmjopen-2020-038503 (PMC7789460; doi:10.1136/bmjopen-2020-038503)

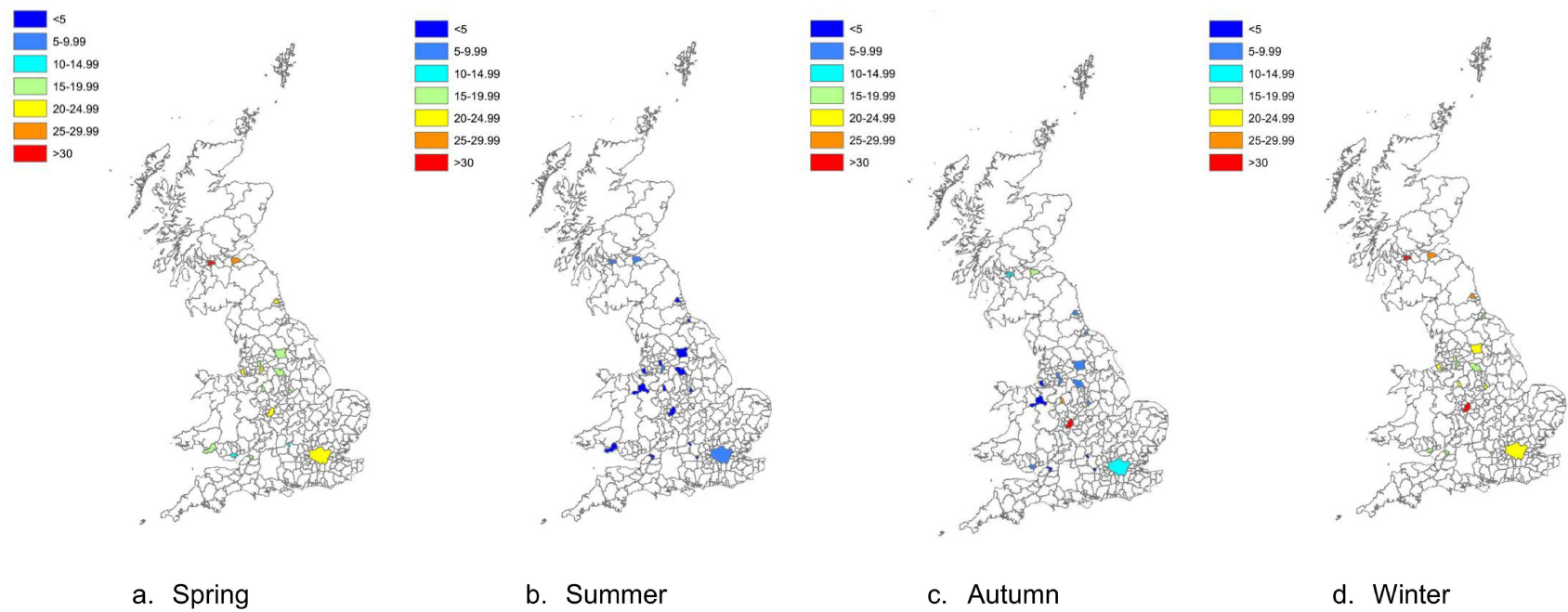

Supplement: Supplementary data [file bmjopen-2020-038503supp001.pdf]
