# Supplementary material for: Distribution of vitamin D status in the UK: a cross-sectional analysis of UK Biobank
Source: BMJ Open. 2021 Jan 6;11(1):e038503. doi: 10.1136/bmjopen-2020-038503 (PMC7789460; doi:10.1136/bmjopen-2020-038503)
Supplement: Author's manuscript [file bmjopen-2020-038503.draft_revisions.pdf]

BMJ Open

BMJ Open is committed to open peer review. As part of this commitment we make the peer review history of every article we publish publicly available.

When an article is published we post the peer reviewers' comments and the authors' responses online. We also post the versions of the paper that were used during peer review. These are the versions that the peer review comments apply to.

The versions of the paper that follow are the versions that were submitted during the peer review process. They are not the versions of record or the final published versions. They should not be cited or distributed as the published version of this manuscript.

BMJ Open is an open access journal and the full, final, typeset and author-corrected version of record of the manuscript is available on our site with no access controls, subscription charges or pay-per-view fees (http://bmjopen.bmj.com).

If you have any questions on BMJ Open's open peer review process please email info.bmjopen@bmj.com

BMJ Open

The distribution of Vitamin D status in the UK: a cross-sectional analysis of UK Biobank

Journal:	*BMJ Open*
Manuscript ID	bmjopen-2020-038503
Article Type:	Original research
Date Submitted by the Author:	13-Mar-2020

Complete List of Authors:	Lin, Liang-Yu; London School of Hygiene and Tropical Medicine Faculty of Epidemiology and Population Health, Department of Non-communicable Disease Epidemiology<br>Smeeth, Liam; London School of Hygiene and Tropical Medicine,<br>Langan, Sinead; London School of Hygiene and Tropical Medicine Faculty of Epidemiology and Population Health, Department of Non-communicable Disease Epidemiology<br>Warren-Gash, Charlotte; London School of Hygiene and Tropical Medicine Faculty of Epidemiology and Population Health, Department of Non-Communicable Disease Epidemiology

Keywords:	EPIDEMIOLOGY, Geographical mapping < TROPICAL MEDICINE, NUTRITION & DIETETICS

SCHOLARONE™
Manuscripts

I, the Submitting Author has the right to grant and does grant on behalf of all authors of the Work (as defined in the below author licence), an exclusive licence and/or a non-exclusive licence for contributions from authors who are: i) UK Crown employees; ii) where BMJ has agreed a CC-BY licence shall apply, and/or iii) in accordance with the terms applicable for US Federal Government officers or employees acting as part of their official duties; on a worldwide, perpetual, irrevocable, royalty-free basis to BMJ Publishing Group Ltd ("BMJ") its licensees and where the relevant Journal is co-owned by BMJ to the co-owners of the Journal, to publish the Work in this journal and any other BMJ products and to exploit all rights, as set out in our licence.

The Submitting Author accepts and understands that any supply made under these terms is made by BMJ to the Submitting Author unless you are acting as an employee on behalf of your employer or a postgraduate student of an affiliated institution which is paying any applicable article publishing charge ("APC") for Open Access articles. Where the Submitting Author wishes to make the Work available on an Open Access basis (and intends to pay the relevant APC), the terms of reuse of such Open Access shall be governed by a Creative Commons licence – details of these licences and which Creative Commons licence will apply to this Work are set out in our licence referred to above.

Other than as permitted in any relevant BMJ Author's Self Archiving Policies, I confirm this Work has not been accepted for publication elsewhere, is not being considered for publication elsewhere and does not duplicate material already published. I confirm all authors consent to publication of this Work and authorise the granting of this licence.

For peer review only

Manuscript title:

The distribution of Vitamin D status in the UK: a cross-sectional analysis of UK Biobank

Authors' full names:

Liang-Yu Lin[1]. LSHTM liang-yu.lin@lshtm.ac.uk

ORCID: https://orcid.org/0000-0003-4720-6738

Liam Smeeth[1]. LSHTM Liam.Smeeth@lshtm.ac.uk

ORCID: https://orcid.org/0000-0002-9168-6022

Sinéad M Langan[1]*. LSHTM Sinead.Langan@lshtm.ac.uk

ORCID: https://orcid.org/0000-0002-7022-7441

Charlotte Warren-Gash[1]*. LSHTM Charlotte.Warren-Gash1@lshtm.ac.uk

ORCID: https://orcid.org/0000-0003-4524-3180

* The senior authors contributed equally to this work

Authors' Affiliations:

1. Faculty of Epidemiology & Population Health, London School of Hygiene and Tropical Medicine, London, UK

# Correspondence to:

Dr Liang-Yu Lin, LSHTM, Keppel Street, London, WC1E 7HT, United Kingdom

Tel: +44 (0) 20 7636 8636 liang-yu.lin@lshtm.ac.uk

For peer review only

Keywords:

Vitamin D, Vitamin D deficiency, Seasons, Geographic locations, Ethnicity, Skin Pigmentation

Word count

1989 words

For peer review only

Abstract

Objective

There are no recent large studies describing the distribution of serum vitamin D levels in the UK. It is important to understand the epidemiology of vitamin D deficiency to inform targeted public health recommendations. This study aimed to investigate the distribution of serum vitamin D levels in a large national cohort.

Design:

A cross-sectional study

Setting:

UK Biobank

Participants:

448,364 participants aged 40 to 69, with measured serum vitamin D levels.

Primary and secondary outcome measures:

We investigated the distribution of serum vitamin D levels among UK Biobank participants. Vitamin D deficiency was defined as a serum 25-hydroxyvitamin D level < 25 nmol/L. We analysed vitamin D levels by demographic characteristics, geographical areas across the UK and different seasons.

Results

Asian (n=4,292/7995, 53.7%) and Black (2,455/7042, 34.9%) participants had a higher proportion of vitamin D deficiency than Caucasian participants (n=50,680/422,683, 12.0%). During the spring and winter, the proportion of vitamin D deficiency was higher across the UK and was higher in the north than in the south.

For peer review only

Conclusions

Vitamin D status varies among different ethnic groups and by season and geographical area within the UK. These findings support the vitamin D supplementation recommendations of Public Health England.

Strengths and limitations of this study

- This study used a large cohort of UK adults in middle and older age.
- The findings provide further evidence to support the nutritional supplementation recommendations of Public Health England.
- The cohort is not nationally representative, so the findings cannot be extrapolated to the general population.
- The response rate to questions on vitamin supplementation was low, which limited the ability to estimate the effect of different nutritional supplements on vitamin D levels.

For peer review only

Introduction

Vitamin D is essential to bone formation. It is mainly produced by the skin after sun exposure, and it can also be obtained from food and supplementation. After production or ingestion, it is further metabolised into 25-hydroxyvitamin D, which can be measured in the blood[1]. Vitamin D plays an important role in calcium and phosphate absorption, and its deficiency impairs bone mineralisation, leading to osteopenia or osteoporosis[1]. Therefore, Public Health England suggests that people older than 4 years of age should take 10 µg (400 IU) of vitamin D daily as a supplement during the winter to support musculoskeletal health[2].

There is no current consensus about the definition of vitamin D deficiency. Using the criteria of a blood 25-hydroxyvitamin D level less than 25 nmol/L, a cohort of UK Caucasians (n=7,437) indicated that in winter and spring, the average prevalence of vitamin D deficiency was 15.5% among people who were 45 years old[3]. Similarly, among adults in the National Diet and Nutrition Survey in the UK (n=3,450), the prevalence of vitamin D deficiency was approximately 24.0% in men and 21.7% in women aged between 19 to 64 years[4]. However, the sizes of these studies are relatively small, and there is no recent large study describing the distribution of vitamin D status in the UK.

The UK Biobank is a prospective cohort that is used to investigate risk factors for major diseases in middle and old age; the cohort participants were assessed for their serum vitamin D levels[5 6]. We aimed to conduct a cross-sectional study using UK Biobank data to report the vitamin D deficiency distribution in the UK according to different demographic, seasonal and regional factors.

For peer review only

Methods

Study population

The UK Biobank was compiled from 2006 to 2010 by recruiting participants throughout the UK. Subjects aged 40-69 years who lived within 40 km of one of the 22 UK Biobank assessment centres and who were registered with the UK National Health Service were eligible to be included in the cohort; approximately 500,000 volunteers were recruited[5 7]. UK Biobank participants received a wide range of examinations, including questionnaires and physical measures, and blood, urine and saliva samples were assayed[6]. Only participants with results for serum vitamin D levels were included in our analyses.

Measurement of covariates

At a participant's first visit to a UK Biobank assessment centre, basic demographic characteristics, sociodemographic factors, environmental and lifestyles factors and the date of assessment were collected using a touchscreen questionnaire[5]. Information such as sex, age, ethnic background, skin colour, smoking status, drinking status and sun exposure were recorded. Physical measurements, including weight, body mass index and waist circumference, were also measured. Furthermore, an additional 24-hour dietary recall online questionnaire was sent to some participants after the end of recruitment to collect information about vitamin and/or mineral supplement use[8].

Defining serum vitamin D levels

Biochemical assays of blood samples collected during the baseline evaluation at the assessment centres were performed[6]. Briefly, serum samples were collected in a

For peer review only

silica clot accelerator tube and stored at -80°C. These samples were later processed in a central laboratory using an automated dispensing system[9]. Serum 25-hydroxy vitamin D levels were measured by chemiluminescence immunoassay (CLIA) (DiaSorin Ltd. LIASON XL, Italy). Quality control and quality assurance processes were also conducted to verify the assay results[10].

Statistical analysis

This study is a cross-sectional study describing the distribution of serum vitamin D levels, so only participants with available measurements of serum vitamin D levels were included. We used the standards of vitamin D deficiency adopted by Public Health England[2 11]. A serum 25-hydroxyvitamin D level <25 nmol/l was coded as “deficiency” and 25-50 nmol/l was coded as “insufficiency.” A 25-hydroxyvitamin D level greater than 50 nmol/l was coded as “sufficiency.” Some continuous variables, such as BMI and waist circumference, were coded as categorical variables following National Institute for Health and Care Excellence (NICE) guidance[12]. According to the NICE guidelines, different BMI and waist circumference standards were applied for Asian and Chinese populations. Self-reported ethnic backgrounds were coded into six groups according to the original questionnaire. Subjects with any other unlisted ethnic group, an unclear ethnic group and those participants who preferred not to answer were assigned as “other ethnic group.” The mean outdoor time was recorded as 0.5 hour/day if it was less than 1 hour. Statistical analysis was carried out using Stata/MP version 15 (StataCorp., USA). The prevalence of vitamin D deficiency was calculated by season and geographical area. The date of blood collection for vitamin D examination was categorised into four seasons, according to the Met Office definition of seasons[13]. Geographical information was obtained from the open data released by the Office for National Statistics[14]. The local authority

For peer review only

132 districts, counties and unitary authorities of 22 UK Biobank assessment centres were used as a proxy for living location. Three of the assessment centres in the London area (Barts, Croydon and Hounslow) were regrouped into “Greater London.” The geographical mapping was plotted using ArcGIS® Desktop10.5 (Esri, USA).

Patient and public involvement:

Patients and or the public were not involved in this retrospective study.

Ethical approval

UK Biobank has obtained ethics approval from its Research Ethics Committee (reference: 11/NW/0382). Our study received ethics approval from the UK Biobank (ID:51265) and from the Research Ethics Committee of the London School of Hygiene and Tropical Medicine (reference: 17158). Our study followed the principles of the Declaration of Helsinki[15].

Results

Comparison of included and excluded participants

We compared the included participants with those who did not have their vitamin D levels tested (**Table 1**). 54,156 participants had missing vitamin D values because the data were not returned, or their values fell outside the reportable range. The distribution of categorical variables was similar across the included and excluded groups. The average age and the mean outdoor time in summer were similar among included and excluded participants, while included participants spent more time outside in winter.

For peer review only

Demographic distribution of vitamin D status

A total of 448,364 participants from the UK Biobank who had their serum vitamin D levels tested were included in our analysis (**Table 2**). The average vitamin D levels in each group were as follows: 19.2 nmol/L in the deficiency group, 37.8 nmol/L in the insufficient group and 67.7 nmol/L in the sufficient group. The demographic characteristics according to vitamin D status are shown in **Table 2**. Asian (n=4,292, 53.7%) and Black (n=2,455, 34.9%) participants had a higher proportion of vitamin D deficiency than white participants (n=50,680, 12.0%). Similar findings are also seen when assessing by skin colours. Among supplement users, less than 10% were vitamin D deficient.

Distribution of vitamin D deficiency prevalence by season and region

We compared the regional prevalence of vitamin D deficiency in different seasons in the UK **(Figure 1)**. In winter and spring, vitamin D deficiency was prevalent across the country, while the prevalence was lower in summer and autumn. Furthermore, in spring, autumn and winter, the prevalence of vitamin D deficiency in the northern part of the UK was higher than that in the southern part of the country.

Discussion

In this cross-sectional study, we found that vitamin D deficiency is more prevalent among participants with Asian and Black ethnic backgrounds. The prevalence of vitamin D deficiency is higher in spring and winter, and more participants living in the north were deficient in vitamin D than those in the southern UK. Due to the larger sample size, our study depicts a more accurate distribution of vitamin D status in the UK.

For peer review only

176 Our findings regarding the demographic characteristics and the regional and
177 seasonal changes in relation to vitamin D status are similar to previous studies[3 4],
178 while our study sample size is larger. Our study provides additional evidence to
179 support the Public Health England recommendation for vitamin D supplementation in
180 winter. Furthermore, vitamin D deficiency is more common among some ethnic
181 groups with darker skin colours, which is also supported by a systematic review[16].
182 Therefore, differences in ethnicity, latitude and season should be considered in
183 future nutritional studies of vitamin D.

184 There are some limitations of this study. First, although the size of the UK Biobank is
185 large, and the ethnicity proportion of UK Biobank is similar to the national census,
186 this cohort is not nationally representative [7]. Our findings cannot be extrapolated to
187 the general population, and the prevalence of vitamin D deficiency found in different
188 areas may not reflect the true prevalence in each region[17]. However, our cross-
189 sectional study can inform further studies on the determinants of vitamin D
190 deficiency. Second, there was limited information about vitamin D supplementation
191 because the questionnaires that gathered this information were only given to select
192 participants. As repeated measurements of the cohort are collected, we expect to
193 perform a more thorough analysis in the future[18].

194 Conclusion

195 A higher proportion of individuals of Asian and Black ethnic backgrounds, as well as
196 people with darker skin, are deficient in vitamin D. Vitamin D deficiency is more
197 common in winter and spring, and its prevalence is higher in the northern UK than in
198 the southern UK. Our study supports the Public Health England recommendation for

For peer review only

taking vitamin D supplementation in winter and for people with Black or Asian ethnic backgrounds.

For peer review only

Tables

Table 1 Comparison of included and excluded participants

Characteristics	Included (N=448,364)	Excluded (N=54,156)
Sex		
Female	240,135 (53.6%)	33,258 (61.4%)
Male	208,229 (46.4%)	20,898 (38.6%)
Average age (SD)	56.5 (8.1)	57.0 (7.8)
BMI[1]		
Underweight	2,299 (0.5%)	327 (0.6%)
Healthy weight	144,521 (32.4%)	15,790 (29.9%)
Overweight	189,492 (42.4%)	22,761 (43.1%)
Obese	110,217 (24.7%)	13,917 (26.4%)
Central obesity[1]		
Not obese	177,278 (39.6%)	18,398 (34.8%)
Central obesity	270,139 (60.4%)	34,545 (65.3%)
Ethnic background		
White	422,683 (94.4%)	49,457 (91.5%)
Mixed	2,589 (0.6%)	320 (0.6%)
Asian	7,995 (1.8%)	1,844 (3.4%)
Black	7,042 (1.6%)	992 (1.8%)
Chinese	1,405 (0.3%)	169 (0.3%)
Other ethnicity[2]	6,055 (1.4%)	1,280 (2.4%)
Skin colour		

For peer review only

Light skin	335,052 (75.9%)	39,523(74.8%)
Olive skin	90,297 (20.5%)	10,622 (20.1%)
Dark skin	16,089 (3.6%)	2,692 (5.1%)
Tobacco smoking		
Non-smoker	400,862 (89.6%)	47,359 (88.3%)
Current-smoker	46,690 (10.4%)	6,289 (11.7%)
Alcohol drinking status		
Never drink	35,489 (7.9%)	5,155 (9.6%)
Drink occasionally	101,338 (22.7%)	12,529 (23.4%)
Drink weekly	219,867 (49.1%)	24,870 (46.4%)
Drink daily	90,699 (20.3%)	11,071 (20.7%)
Mean outdoors time in summer (SD)	3.8 (2.4)	3.8 (2.4)
Mean outdoors time in winter (SD)	2.0 (1.8)	1.9(1.7)
Supplementation use		
Vitamin D supplement use	5,541	649
Multivitamin use	16,183	1,593
Fish oil or Calcium use	56,978	6,227

1. The classification is suggested by NICE guidelines; 2. Includes any other unlisted ethnic groups, unclear ethnic groups and participants prefer not to answer;

For peer review only

Table 2 Basic characteristics of the study population

Characteristics	Vitamin D Deficiency (< 25 nmol/L)	Vitamin D Insufficiency (25 – 50 nmol/L)	Vitamin D Sufficiency (> 50 nmol/L)
Serum vitamin D levels (SD)	19.2 (3.9)	37.8 (7.1)	67.7 (14.5)
Sex			
Female	32,042 (13.3%)	100,428 (41.8%)	107,665 (44.8%)
Male	28,391 (13.6%)	87,371 (42.0%)	92,467 (44.4%)
Age (SD)	54.7 (8.1)	56.2 (8.1)	57.3 (8.0)
BMI[1]			
Underweight	421 (18.3%)	844 (36.7%)	1,034 (45%)
Healthy weight	15,810 (10.9%)	54,117 (37.5%)	74,594 (51.6%)
Overweight	22,743 (12%)	79,355 (41.9%)	87,394 (46.1%)
Obese	20,912 (19.0%)	52,705 (47.8%)	36,600 (33.2%)
Central obesity[1]			
Non-obese	19,435 (11.0%)	67,002 (37.8%)	90,841 (51.2%)
Central obesity	40,728 (15.1%)	120,393 (44.6%)	109,018 (40.4%)
Ethnic background			
White	50,680 (12.0%)	176,203 (41.7%)	195,800 (46.3%)
Mixed	642 (24.8%)	1,241 (47.9%)	706 (27.3%)
Asian	4,292 (53.7%)	2,979 (37.3 %)	724 (9.1%)
Black	2,455 (34.9%)	3,503 (49.7%)	1,084 (15.4%)
Chinese	381 (27.1%)	787 (56.0%)	237 (16.9%)
Other ethnicity[2]	1,855 (30.6%)	2,816 (46.5%)	1,384 (22.9%)

For peer review only

Skin colour			
Light skin	43,034 (12.8%)	142,451 (42.5%)	149,567 (44.6%)
Olive skin	9,868 (10.9%)	35,331 (39.1%)	45,098 (49.9%)
Dark skin	6,113 (38.0%)	7,120 (44.3%)	2,856 (17.8%)
Tobacco smoking			
Non-smoker	50,048 (12.5%)	167,504 (41.8%)	183,310 (45.7%)
Current-smoker	10,138 (21.7%)	19,944 (42.7%)	16,608 (35.6%)
Alcohol drinking status			
Never drink	8,402 (23.7%)	15,267 (43.0%)	11,820 (33.3%)
Drink occasionally	16,593 (16.4%)	45,210 (44.6%)	39,535 (39.0%)
Drink weekly	24,447 (11.1%)	90,839 (41.3%)	104,581 (47.6%)
Drink daily	10,685 (11.8%)	36,064 (39.8%)	43,950 (48.5%)
Mean outdoors time in summer (hours) (SD)	3.3 (2.4)	3.6 (2.4)	4.1 (2.4)
Mean outdoors time in winter (hours) (SD)	1.7 (1.8)	1.9 (1.8)	2.1 (1.8)
Supplementation use			
Vitamin D supplement (N=5541)	594 (10.7%)	2,213 (39.9%)	2,734 (49.3%)
Multivitamin supplement (N=16,183)	1,487 (9.1%)	6,465 (40.0%)	8,231 (50.9%)
Fish oil or Calcium supplement (N=56,978)	3,262 (5.7%)	19,319 (33.9%)	34,397 (60.4%)

1. The classification is suggested by NICE guidelines; 2. Includes any other unlisted ethnic groups, unclear ethnic groups and participants prefer not to answer.

For peer review only

Authors' contribution

LYL contributed to the design of the study, analysed the data, drafted and revised the manuscript according to other authors' comments; CWG contributed to the design of the study, made critical comments on the manuscript and revised the paper critically; SML contributed to the design of the study, made critical comments on the manuscript and revised the paper critically; LS contributed to the design of the study and revised the paper critically. All authors approved the final version of the protocol.

Competing interest:

None declared.

Funding statement:

LYL is funded by the scholarship of government sponsorship for overseas study by the Ministry of Education Republic of China (Taiwan). CWG is supported by a Wellcome Intermediate Clinical Fellowship (201440_Z_16_Z). SML is funded by a Wellcome Senior Clinical Fellowship in Science (205039/Z/16/Z).

Data sharing statement:

Other researchers can apply for UK Biobank data to answer specific research questions. We will upload our analysis codes to LSHTM Data Compass, an open data repository for research outputs from LSHTM (https://datacompass.lshtm.ac.uk/).

Acknowledgement

We thank Yi-Tin Ching for assistance with technical support of ArcGIS mapping.

Patient consent for publication:

Not required

For peer review only

For peer review only - http://bmjopen.bmj.com/site/about/guidelines.xhtml

References:

1. Holick MF. Vitamin D deficiency. *The New England journal of medicine* 2007;357(3):266-81. doi: 10.1056/NEJMra070553 [published Online First: 2007/07/20]
2. The Scientific Advisory Committee on Nutrition. Vitamin D and Health: Public Health England 2016.
3. Hypponen E, Power C. Hypovitaminosis D in British adults at age 45 y: nationwide cohort study of dietary and lifestyle predictors. *The American journal of clinical nutrition* 2007;85(3):860-8. doi: 10.1093/ajcn/85.3.860 [published Online First: 2007/03/09]
4. Public Health England. Results from Years 1, 2, 3 and 4 (combined) of the Rolling Programme (2008/2009 – 2011/2012) In: Beverley Bates AL, Ann Prentice, Chris Bates, Polly Page, Sonja Nicholson and Gillian Swan, ed. National Diet and Nutrition Survey (NDNS) London: Public Health England, 2014:116.
5. Sudlow C, Gallacher J, Allen N, et al. UK biobank: an open access resource for identifying the causes of a wide range of complex diseases of middle and old age. *PLoS Med* 2015;12(3):e1001779. doi: 10.1371/journal.pmed.1001779 [published Online First: 2015/04/01]
6. UK Biobank. UK Biobank Biomarker Panel, 2015.
7. Fry A, Littlejohns TJ, Sudlow C, et al. Comparison of Sociodemographic and Health-Related Characteristics of UK Biobank Participants With Those of the General Population. *American journal of epidemiology* 2017;186(9):1026-34. doi: https://dx.doi.org/10.1093/aje/kwx246
8. UK Biobank. Online 24-hour dietary recall questionnaire, 2012.
9. UK Biobank. Biomarker assay quality procedures: approaches used to minimise systematic and random errors. 1.2 ed, 2019.
10. UK Biobank. UK Biobank Biomarker Project. 1 ed, 2019.
11. Pearce SH, Cheetham TD. Diagnosis and management of vitamin D deficiency. *Bmj* 2010;340:b5664. doi: 10.1136/bmj.b5664
12. National Institute for Health and Care Excellence (NICE). BMI: preventing ill health and premature death in black, Asian and other minority ethnic groups: National Institute for Health and Clinical Excellence 2013.
13. Met Office. Seasons 2019 [Available from: https://www.metoffice.gov.uk/weather/learn-about/weather/seasons accessed 25/11 2019.
14. Office for National Statistics. Counties and Unitary Authorities Full Clipped Boundaries in England and Wales, 2018.
15. World Medical A. World Medical Association Declaration of Helsinki: ethical principles for medical research involving human subjects. *JAMA* 2013;310(20):2191-94. doi: 10.1001/jama.2013.281053
16. Renzaho AMN, Halliday JA, Nowson C. Vitamin D, obesity, and obesity-related chronic disease among ethnic minorities: A systematic review. *Nutrition* 2011;27(9):868-79. doi: https://doi.org/10.1016/j.nut.2010.12.014
17. UK Biobank. Access matter: representativeness of the UK Biobank resource 2017 [Available from: http://www.ukbiobank.ac.uk/wp-content/uploads/2017/03/access-matters-representativeness-1.pdf accessed 16/05 2019.
18. UK Biobank. Provisional timeline for future data availability 2019 [Available from: http://biobank.ndph.ox.ac.uk/showcase/exinfo.cgi?src=future_timelines accessed 28/11 2019.

For peer review only

For peer review only - http://bmjopen.bmj.com/site/about/guidelines.xhtml

Figures

Figure 1 Mean percentage of participants with vitamin D deficiency by UK Biobank assessment centres and season

For peer review only

19

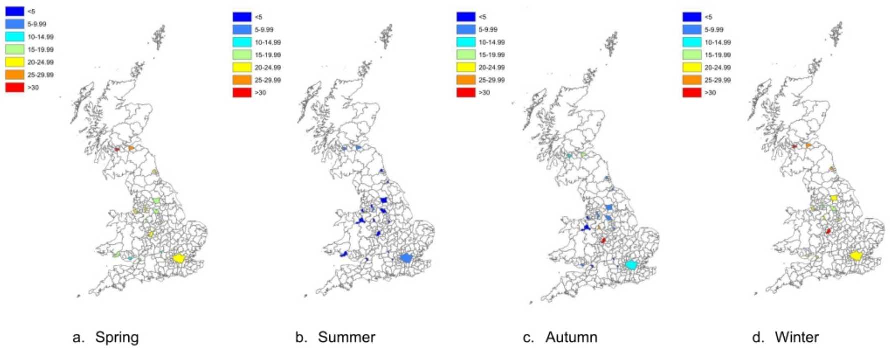

Figure 1 Mean percentage of participants with vitamin D deficiency by UK Biobank assessment centres and season

STROBE 2007 (v4) checklist of items to be included in reports of observational studies in epidemiology*
Checklist for cohort, case-control, and cross-sectional studies (combined)

Section/Topic	Item #	Recommendation	Reported on page #
**Title and abstract**	1	(*a*) Indicate the study's design with a commonly used term in the title or the abstract	#1
		(*b*) Provide in the abstract an informative and balanced summary of what was done and what was found	#4
**Introduction**			
Background/rationale	2	Explain the scientific background and rationale for the investigation being reported	#6
Objectives	3	State specific objectives, including any pre-specified hypotheses	#6
**Methods**			
Study design	4	Present key elements of study design early in the paper	#8
Setting	5	Describe the setting, locations, and relevant dates, including periods of recruitment, exposure, follow-up, and data collection	#7
Participants	6	(*a*) *Cohort study*—Give the eligibility criteria, and the sources and methods of selection of participants. Describe methods of follow-up<br>*Case-control study*—Give the eligibility criteria, and the sources and methods of case ascertainment and control selection. Give the rationale for the choice of cases and controls<br>*Cross-sectional study*—Give the eligibility criteria, and the sources and methods of selection of participants	#7
		(*b*) *Cohort study*—For matched studies, give matching criteria and number of exposed and unexposed<br>*Case-control study*—For matched studies, give matching criteria and the number of controls per case	Not applicable
Variables	7	Clearly define all outcomes, exposures, predictors, potential confounders, and effect modifiers. Give diagnostic criteria, if applicable	#7
Data sources/ measurement	8*	For each variable of interest, give sources of data and details of methods of assessment (measurement). Describe comparability of assessment methods if there is more than one group	#7
Bias	9	Describe any efforts to address potential sources of bias	#8
Study size	10	Explain how the study size was arrived at	#7

For peer review only

Quantitative variables	11	Explain how quantitative variables were handled in the analyses. If applicable, describe which groupings were chosen and why	#7
Statistical methods	12	(*a*) Describe all statistical methods, including those used to control for confounding	#8
		(*b*) Describe any methods used to examine subgroups and interactions	Not applicable
		(*c*) Explain how missing data were addressed	Not applicable
		(*d*) *Cohort study*—If applicable, explain how loss to follow-up was addressed<br>*Case-control study*—If applicable, explain how matching of cases and controls was addressed<br>*Cross-sectional study*—If applicable, describe analytical methods taking account of sampling strategy	Not applicable
		(*e*) Describe any sensitivity analyses	Not applicable
**Results**			
Participants	13*	(a) Report numbers of individuals at each stage of study—eg numbers potentially eligible, examined for eligibility, confirmed eligible, included in the study, completing follow-up, and analysed	#9
		(b) Give reasons for non-participation at each stage	#9
		(c) Consider use of a flow diagram	
Descriptive data	14*	(a) Give characteristics of study participants (eg demographic, clinical, social) and information on exposures and potential confounders	#10
		(b) Indicate number of participants with missing data for each variable of interest	#13
		(c) *Cohort study*—Summarise follow-up time (eg, average and total amount)	Not applicable
Outcome data	15*	*Cohort study*—Report numbers of outcome events or summary measures over time	Not applicable
		Case-control study—Report numbers in each exposure category, or summary measures of exposure	Not applicable
		Cross-sectional study—Report numbers of outcome events or summary measures	#10, #15
Main results	16	(*a*) Give unadjusted estimates and, if applicable, confounder-adjusted estimates and their precision (eg, 95% confidence interval). Make clear which confounders were adjusted for and why they were included	Not applicable
		(*b*) Report category boundaries when continuous variables were categorized	#15

For peer review only

		(*c*) If relevant, consider translating estimates of relative risk into absolute risk for a meaningful time period	Not applicable
Other analyses	17	Report other analyses done—eg analyses of subgroups and interactions, and sensitivity analyses	Not applicable
**Discussion**			
Key results	18	Summarise key results with reference to study objectives	#10
Limitations	19	Discuss limitations of the study, taking into account sources of potential bias or imprecision. Discuss both direction and magnitude of any potential bias	#11
Interpretation	20	Give a cautious overall interpretation of results considering objectives, limitations, multiplicity of analyses, results from similar studies, and other relevant evidence	#11
Generalisability	21	Discuss the generalisability (external validity) of the study results	#12
**Other information**			
Funding	22	Give the source of funding and the role of the funders for the present study and, if applicable, for the original study on which the present article is based	#2

*Give information separately for cases and controls in case-control studies and, if applicable, for exposed and unexposed groups in cohort and cross-sectional studies.

Note: An Explanation and Elaboration article discusses each checklist item and gives methodological background and published examples of transparent reporting. The STROBE checklist is best used in conjunction with this article (freely available on the Web sites of PLoS Medicine at http://www.plosmedicine.org/, Annals of Internal Medicine at http://www.annals.org/, and Epidemiology at http://www.epidem.com/). Information on the STROBE Initiative is available at www.strobe-statement.org.

For peer review only

BMJ Open

The distribution of Vitamin D status in the UK: a cross-sectional analysis of UK Biobank

Journal:	*BMJ Open*
Manuscript ID	bmjopen-2020-038503.R1
Article Type:	Original research
Date Submitted by the Author:	06-Nov-2020
Complete List of Authors:	Lin, Liang-Yu; London School of Hygiene and Tropical Medicine Faculty of Epidemiology and Population Health, Department of Non-communicable Disease Epidemiology<br>Smeeth, Liam; London School of Hygiene and Tropical Medicine,<br>Langan, Sinead; London School of Hygiene and Tropical Medicine Faculty of Epidemiology and Population Health, Department of Non-communicable Disease Epidemiology<br>Warren-Gash, Charlotte; London School of Hygiene and Tropical Medicine Faculty of Epidemiology and Population Health, Department of Non-Communicable Disease Epidemiology
**Primary Subject Heading**:	Epidemiology
Secondary Subject Heading:	Nutrition and metabolism
Keywords:	EPIDEMIOLOGY, NUTRITION & DIETETICS, PUBLIC HEALTH

SCHOLARONE™ Manuscripts

I, the Submitting Author has the right to grant and does grant on behalf of all authors of the Work (as defined in the below author licence), an exclusive licence and/or a non-exclusive licence for contributions from authors who are: i) UK Crown employees; ii) where BMJ has agreed a CC-BY licence shall apply, and/or iii) in accordance with the terms applicable for US Federal Government officers or employees acting as part of their official duties; on a worldwide, perpetual, irrevocable, royalty-free basis to BMJ Publishing Group Ltd ("BMJ") its licensees and where the relevant Journal is co-owned by BMJ to the co-owners of the Journal, to publish the Work in this journal and any other BMJ products and to exploit all rights, as set out in our licence.

The Submitting Author accepts and understands that any supply made under these terms is made by BMJ to the Submitting Author unless you are acting as an employee on behalf of your employer or a postgraduate student of an affiliated institution which is paying any applicable article publishing charge ("APC") for Open Access articles. Where the Submitting Author wishes to make the Work available on an Open Access basis (and intends to pay the relevant APC), the terms of reuse of such Open Access shall be governed by a Creative Commons licence – details of these licences and which Creative Commons licence will apply to this Work are set out in our licence referred to above.

Other than as permitted in any relevant BMJ Author's Self Archiving Policies, I confirm this Work has not been accepted for publication elsewhere, is not being considered for publication elsewhere and does not duplicate material already published. I confirm all authors consent to publication of this Work and authorise the granting of this licence.

For peer review only

Manuscript title:

The distribution of Vitamin D status in the UK: a cross-sectional analysis of UK Biobank

Authors' full names:

Liang-Yu Lin[1]. LSHTM liang-yu.lin@lshtm.ac.uk

ORCID: https://orcid.org/0000-0003-4720-6738

Liam Smeeth[1]. LSHTM Liam.Smeeth@lshtm.ac.uk

ORCID: https://orcid.org/0000-0002-9168-6022

Sinéad M Langan[1]*. LSHTM Sinead.Langan@lshtm.ac.uk

ORCID: https://orcid.org/0000-0002-7022-7441

Charlotte Warren-Gash[1]*. LSHTM Charlotte.Warren-Gash1@lshtm.ac.uk

ORCID: https://orcid.org/0000-0003-4524-3180

* The senior authors contributed equally to this work

Authors' Affiliations:

1. Faculty of Epidemiology & Population Health, London School of Hygiene and Tropical Medicine, London, UK

# Correspondence to:

Dr Liang-Yu Lin, LSHTM, Keppel Street, London, WC1E 7HT, United Kingdom

Tel: +44 (0) 20 7636 8636 liang-yu.lin@lshtm.ac.uk

For peer review only

Keywords:

Vitamin D, Vitamin D deficiency, Seasons, Geographic locations, Ethnicity

Word count: 2780

For peer review only

For peer review only - http://bmjopen.bmj.com/site/about/guidelines.xhtml

ABSTRACT

Objective

No recent large studies have described the distribution of vitamin D status in the UK. Understanding the epidemiology of vitamin D deficiency is important to inform targeted public health recommendations. This study aimed to investigate the distribution of factors associated with serum vitamin D status in a large national cohort.

Design

A cross-sectional study.

Setting

The UK Biobank, a prospective cohort study following the health and wellbeing of middle-aged and older adults recruited between 2006 and 2010.

Participants

A total of 449,943 participants aged 40 to 69 years with measured serum vitamin D status were eligible for the analysis. Participants completed a questionnaire about sex, age, ethnic background, vitamin D supplementation, smoking, drinking and socioeconomic status.

Primary and secondary outcome measures

We investigated the distribution of serum vitamin D status and the association between demographic factors and vitamin D deficiency or insufficiency. Vitamin D deficiency was defined as a serum 25-hydroxyvitamin D level <25 nmol/L.

For peer review only

Multivariable logistic regression was used to assess the association between demographic factors and vitamin D status.

Results

Asian (n=4,297/8000, 53.7%) and Black (n=2,459/7046, 34.9%) participants had a higher proportion of vitamin D deficiency than white participants (n=50,920/422,907, 12%). During spring and winter, the proportion of vitamin D deficiency was higher across the UK and higher in the north than in the south. Male sex, abnormal body mass index, non-white ethnic backgrounds, smoking, and being more socioeconomically deprived were associated with higher odds of vitamin D deficiency. Older age, taking vitamin D supplements and drinking alcohol were associated with lower odds of deficiency.

Conclusions

Vitamin D status varied among different ethnic groups and by season and geographical area within the UK. Taking supplements was associated with a lower risk of vitamin D deficiency. These findings support the vitamin D supplementation recommendations of Public Health England.

Abstract words count: 300

For peer review only

61 **Strengths and limitations of this study**

62 • This study used a large cohort of British adults in middle and older age, and its large sample size provides more statistical power in assessing associations.

65 • The findings provide further evidence to support the nutritional supplementation recommendations of Public Health England.

67 • The cohort is not nationally representative, so the findings cannot be extrapolated to the general population.

69 • The questionnaire on vitamin supplementation was self-reported and thus cannot be used to estimate the required supplement amount.

For peer review only

For peer review only - http://bmjopen.bmj.com/site/about/guidelines.xhtml

INTRODUCTION

Vitamin D is essential to bone formation. It is mainly produced by the skin after sun exposure, and it can also be obtained from food and supplementation. After production or ingestion, it is further metabolised in the liver into 25-hydroxyvitamin D, which can be measured in the blood. 25-hydroxyvitamin D is further transformed in the kidneys into 1,25-dihydroxyvitamin D, the active form of vitamin D. This active vitamin D metabolite acts on the intestines and kidneys, regulates the absorption and excretion of calcium and phosphate, and facilitates the mineralisation of bone. Vitamin D deficiency may impair bone mineralisation, leading to osteopenia or osteoporosis[1]. Currently, Public Health England suggests that people older than 4 years of age should take 10 μg (400 IU) of vitamin D daily as a supplement during the winter to support musculoskeletal health[2].

In addition to the classical effects of mineral homeostasis, more recent attention has focused on novel effects of vitamin D. Previous studies have indicated that vitamin D may have potential immunomodulatory effects. Active vitamin D can enhance innate immunity by increasing the production of antimicrobial peptides, and vitamin D can regulate adaptive immunity as well[3]. Epidemiological studies have also indicated that vitamin D is associated with autoimmune diseases such as inflammatory bowel disease, type I diabetes mellitus and multiple sclerosis[4]. In addition, a meta-analysis using original data from trials suggested that taking vitamin D supplementation may decrease the risk of respiratory infections[5].

Although 1,25-dihydroxyvitamin D plays an active role in metabolism, its half-life is less than 4 hours, while the half-life of 25-hydroxyvitamin D is around 2–3 weeks. Thus, clinical 25-hydroxyvitamin D levels in the blood have been used to assess vitamin D status[6]. 25-hydroxyvitamin D can be analysed using either

For peer review only - http://bmjopen.bmj.com/site/about/guidelines.xhtml

chemiluminescence immunoassay or tandem mass spectrometry, which are recognised by the Royal Osteoporosis Society and Public Health England[6 7]. However, no current consensus exists about the definition of vitamin D deficiency, so each study may use different standards. The Endocrine Society of the US defined vitamin D deficiency as 25-hydroxyvitamin D below 50 nmol/L, while the criterion of Public Health England is 25-hydroxyvitamin D less than 25 nmol/L.

Using the criterion of a blood vitamin D level less than 25 nmol/L, a cohort of British Caucasians (n=7,437) indicated that in winter and spring, the average prevalence of vitamin D deficiency was 15.5% among people who were 45 years old[8]. Similarly, among adults in the National Diet and Nutrition Survey in the UK (n=3,450), the prevalence of vitamin D deficiency was approximately 24.0% in men and 21.7% in women aged between 19 and 64 years[9]. However, these studies were relatively small, and no recent large study has described the distribution of vitamin D status in the UK.

The UK Biobank is a prospective, nationwide cohort used to investigate risk factors for major diseases in middle and old age; cohort participants were assessed for various biochemical biomarkers, including serum vitamin D status[10 11]. By using UK Biobank data, we aimed to conduct a cross-sectional study to investigate different demographic, seasonal and regional factors associated with vitamin D deficiency distribution in the UK.

METHODS

Study population

The UK Biobank was compiled from 2006 to 2010 by recruiting participants throughout the UK. People aged 40 to 69 years who lived within 40 km of one of the

For peer review only

For peer review only - http://bmjopen.bmj.com/site/about/guidelines.xhtml

22 UK Biobank assessment centres and who were registered with the UK National Health Service were eligible to be included in the cohort; approximately 500,000 volunteers were recruited[10 12]. UK Biobank participants received a wide range of examinations, including questionnaires and physical measures, and blood, urine and saliva samples were assayed[6]. Only participants with results for serum vitamin D status were included in our analyses.

Measurement of covariates

At each participant's first visit to a UK Biobank assessment centre, a touchscreen questionnaire collected basic demographic characteristics; sociodemographic, environmental and lifestyle factors; and the date of assessment[10]. Information such as sex, age, ethnic background, skin colour, smoking status, drinking status, sun exposure, and vitamin and mineral supplement use were recorded. Physical measurements, including body mass index (BMI) and waist circumference were also taken. The index of multiple deprivation (IMD) of participants was obtained through data linkage[13].

Defining serum vitamin D status

Biochemical assays were performed on blood samples collected during the baseline evaluation at the assessment centres [6]. Briefly, serum samples were collected in a silica clot accelerator tube and stored at -80°C. These samples were later processed in a central laboratory using an automated dispensing system[14]. Serum 25-hydroxyvitamin D status was measured by chemiluminescence immunoassay (DiaSorin Ltd. LIASON XL, Italy), which was certified by the Vitamin D Standardization-Certification Program of the Centers for Disease Control and Prevention[15]. To ensure the precision of analysis, quality control samples at different

For peer review only - http://bmjopen.bmj.com/site/about/guidelines.xhtml

144 concentrations were analysed[16], and the testing assay for vitamin D was verified through the RIQAS Immunoassay Speciality I EQA programme (Randox Laboratories Ltd.), an external quality assurance scheme[17].

Statistical analysis

This was a cross-sectional study describing the distribution of serum vitamin D status, so only participants who had at least one available measurement of serum vitamin D status were included. We used the standards of vitamin D deficiency adopted by Public Health England[6 18]. A serum 25-hydroxyvitamin D level less than 25 nmol/L was coded as “deficiency”, and 25-50 nmol/L was coded as “insufficiency”. A 25-hydroxyvitamin D level greater than 50 nmol/L was coded as “sufficiency”. Vitamin D was coded as missing in cases of no reportable value, values above or below the reportable limits, or unrecoverable aliquot problems.

To compare the distribution of vitamin D status by these factors associated with vitamin D, continuous covariates, such as BMI and waist circumference, were coded as categorical variables following National Institute for Health and Care Excellence (NICE) guidance[19]. According to the guidelines, different BMI and waist circumference standards were applied for Asian and Chinese populations. Self-reported ethnic backgrounds were coded into six groups according to the original questionnaire. Participants with any other unlisted ethnic group, with an unclear ethnic group or preferring not to answer were assigned “other ethnic group”. The mean outdoor time was recorded as 0.5 hours/day if it was less than 1 hour. Responses regarding vitamin and mineral use were further regrouped as “vitamin D, multivitamin, fish oil and calcium use” and “other vitamin and mineral use”. IMD scores were categorised into five quintiles, with the fifth quintile assigned the ”most deprived” group. The locations of 22 Biobank assessment centres were grouped

For peer review only - http://bmjopen.bmj.com/site/about/guidelines.xhtml

using the geographical regions of the UK[20]. The date of blood collection for vitamin D examination was categorised into four seasons, according to the Met Office definitions[21].

Demographic factors including sex, age, ethnicity, BMI, smoking, drinking alcohol, IMD, vitamin D testing season, and geographical location were further analysed in terms of their association with vitamin D insufficiency (<50 nmol/L) and deficiency (<25 nmol/L) using simple and multivariable logistic regression models. Covariates such as central obesity were not included in the model to avoid probable collinearity with BMI. All statistical analysis was carried out using Stata/MP version 15 (StataCorp, USA).

Patient and public involvement

Patients and/or the public were not involved in this retrospective study.

Ethical approval

The UK Biobank obtained ethics approval from its Research Ethics Committee (reference: 11/NW/0382). Our study received ethics approval from the UK Biobank (ID:51265) and the Research Ethics Committee of the London School of Hygiene and Tropical Medicine (reference: 17158). Our study followed the principles of the Declaration of Helsinki[22].

RESULTS

Comparison of included and excluded participants

We compared the included participants with those who did not have their vitamin D status tested (**Table 1**). Vitamin D values were missing for 52,550 participants, who were excluded. Among excluded participants, the proportion of female participants (61.7%) was slightly higher than among the included participants (53.6%), while the

For peer review only - http://bmjopen.bmj.com/site/about/guidelines.xhtml

distribution of the other categorical variables was similar across the included and excluded groups. The average age and mean outdoor time in summer were similar among included and excluded participants, while included participants spent more time outside in winter.

Demographic distribution of vitamin D status

The demographic characteristics according to vitamin D status are shown in **Table 2**. The average vitamin D levels were 19.2 nmol/L in the deficient group, 37.8 nmol/L in the insufficient group and 67.7 nmol/L in the sufficient group. The distribution of vitamin D status was similar in both sexes, and the mean age of the vitamin D sufficiency group was older than other groups. The proportion of vitamin deficiency was lower among people with healthy weight (n=15,886, 11%) and non-obese people (n=177,348, 11%). Asian (n=4,297, 53.7%) and Black (n=2,459, 34.9%) people had a higher proportion of vitamin D deficiency than white participants (n=50,920, 12.0%). Vitamin D deficiency was more prevalent among current smokers (n=10,170, 21.7%) and those who never drank alcohol (n=8,419, 23.7%). People deficient in vitamin D spent less outdoor time in winter (mean = 1.7 hours). The proportion of vitamin D deficiency was lower among participants taking vitamin D and associated mineral supplements (n=11,368, 7%) compared with people taking any other supplements (n= 48,877, 17%). The most deprived people had the highest proportion of vitamin D deficiency (n=17,602, 20.4%), while the least deprived participants had the lowest deficiency proportion (n=8,414, 9.5%).
Vitamin D deficiency was more common in winter (n=21,066, 23.1%) and spring (25,912, 20%). Participants who visited assessment centres in Scotland had the highest proportion of vitamin D deficiency (n=7,618, 23.5%) compared with other assessment centres in the southern UK (**Table 2**). A map showing the distribution of

For peer review only - http://bmjopen.bmj.com/site/about/guidelines.xhtml

the proportion of vitamin D deficiency in different seasons by each UK Biobank assessment centre is illustrated in **Supplementary Figure 1**. This shows that in winter and spring, vitamin D deficiency was prevalent across the country, while in summer and autumn it was less common. Furthermore, in spring, autumn and winter, the proportion of vitamin D deficiency in the northern part of the UK was higher than in the southern part of the country (**Supplementary Figure 1**).

Association between demographic factors and vitamin D status

To assess the association of factors related to vitamin D status, the odds ratio of having vitamin D deficiency (25(OH)D <25 nmol/L) or insufficiency (25(OH)D <50 nmol/L) were summarised in **Table 3**. Male sex, abnormal BMI, smoking, non-white ethnicity, and being more deprived had greater odds of vitamin D deficiency or insufficiency. Older age, drinking alcohol, and taking vitamin D, multivitamin, fish oil or calcium supplements were associated with lower odds of vitamin D deficiency or insufficiency. Compared with summer, receiving testing in spring or winter had greater odds of vitamin D deficiency or insufficiency. For the regions with assessment centres, Scotland had higher odds of vitamin D deficiency and insufficiency compared with other regions in the UK. After adjusting for the variables listed in Table 3, these associations remained (**Table 3**).

For peer review only

DISCUSSION

In this cross-sectional study, we found that vitamin D deficiency was more prevalent among people of colour, in spring and winter, and in the northern UK. Male sex, abnormal BMI, non-white ethnic background, smoking and being more deprived increased the odds of vitamin D deficiency. Taking vitamin D supplements, drinking alcohol, and older age were associated with decreased odds of vitamin D deficiency.

For peer review only - http://bmjopen.bmj.com/site/about/guidelines.xhtml

242 Our study showed that vitamin D status was strongly associated with seasonality and
243 geographical distribution, which is similar to previous studies[8 9]. Other studies have
244 also indicated that vitamin D deficiency is more prevalent among non-white ethnic
245 groups, such as south Asians. A small longitudinal study (n=140) in the UK showed
246 that more than 90% of south Asian women in the cohort had vitamin D insufficiency
247 (25-hydroxyvitamin D <50 nmol/L) throughout the year, while the proportion of
248 insufficiency was much lower in the white comparison groups (20.3% in summer and
249 70% in winter)[23]. A systematic review of 19 cross-sectional studies and one cohort
250 study indicated that serum hydroxyvitamin D levels were lower among Black, African
251 American and non-Hispanic Black groups, compared with Caucasian groups [24].
252 These findings indicate the importance of differences in ethnicity, latitude and
253 season, which should always be considered in dietary guidelines and future
254 nutritional studies of vitamin D.

255 We found that abnormal BMI, tobacco smoking, and deprivation increased the odds
256 of vitamin D deficiency or insufficiency, while taking vitamin D and associated
257 mineral supplements was associated with a lower risk of vitamin D deficiency or
258 insufficiency. These results are consistent with other research about factors
259 associated with vitamin D[1 25 26]. The common vitamin D supplements contain around
260 400 to 2,000 IU of vitamin D_2 or D_3, meeting the official daily intake guide. Our
261 findings provide some evidence about the effect of vitamin D supplements as well as
262 the Public Health England recommendation for vitamin D supplementation in winter
263 and for some ethnic backgrounds[2].

264 Curiously, age and drinking alcohol were inversely associated with vitamin D status.
265 Aging has been regarded as a risk factor for vitamin D deficiency; however, our
266 results showed older age slightly decreased the odds of deficiency. A possible

For peer review only - http://bmjopen.bmj.com/site/about/guidelines.xhtml

explanation for this is that our study population focused on middle-aged or older participants, who may be more likely to receive prescriptions containing vitamin D. However, this information was not included in our study. Regarding drinking alcohol, although a previous systematic review reported no consistent correlation between alcohol intake and serum vitamin D levels[27], recent studies have shown a negative association. A study using the National Health and Nutrition Examination Survey (NHANES) database in the US from 2001 to 2010 showed the prevalence of vitamin D deficiency among current alcohol drinkers was 38% lower than in non-drinkers[26]. Similarly, a cross-sectional survey in Portugal among 1,500 participants aged over 65 years showed that alcohol drinkers had lower odds of vitamin D deficiency compared with non-drinkers (moderate drinker: OR=0.49 [CI: 0.32 to 0.73]; excessive drinker: OR=0.48 [CI: 0.27 to 0.85])[25]. The mechanism behind this possible association is still unclear, and future studies about vitamin D should take alcohol into account.

The key strengths of our study are its large sample size and wide range of measurements, which provide more statistical power in analysing the factors associated with vitamin D deficiency. Moreover, information about the exposure and outcomes was collected following a pre-defined protocol, and samples were processed systematically, which minimises potential differential or non-differential misclassification bias. Our analysis about the association between demographic factors and vitamin D deficiency is solid. However, several important limitations need to be considered. Despite the large size of the UK Biobank, this cohort is not nationally representative. Compared with non-participants, the participants of the UK Biobank were more likely to be older, female, and with a higher socioeconomic status and fewer health conditions[12]. Due to this healthy volunteer effect, our findings

For peer review only - http://bmjopen.bmj.com/site/about/guidelines.xhtml

on vitamin D status cannot be generalised to the UK population, and we may have underestimated the prevalence of vitamin D deficiency. Nevertheless, as the healthy volunteer effect does not affect the validity of exposure–outcome relationships, our findings on the factors associated with vitamin D status are likely to be valid.

This analysis has concentrated on vitamin D status using serum vitamin D levels obtained from a single blood test, which may not reflect long-term vitamin D status. Additionally, the questionnaires collecting information about vitamin D supplementation and outdoor physical activity could not precisely quantify the exposure. In further research, a more representative sample should be considered to assess the prevalence of vitamin D deficiency in the UK. Researchers may consider using repeated measurements to assess long-term vitamin D status more precisely or including clinical diagnosis or vitamin D prescription to capture clinical status. In addition, collecting information to quantify vitamin D supplementation and outdoor activities in different seasons can further validate the recommendation of Public Health England.

For peer review only

CONCLUSION

Vitamin D deficiency was more common in winter and spring, and its prevalence was higher in the northern UK than the southern UK. Male sex, abnormal BMI, Asian and Black ethnic backgrounds, and tobacco smoking were associated with higher odds of vitamin D deficiency. Taking vitamin D supplements and drinking alcohol were associated with lower odds of vitamin D deficiency. These results provide some evidence supporting the Public Health England recommendation for taking vitamin D supplementation in winter and for people with Black or Asian ethnic backgrounds.

For peer review only - http://bmjopen.bmj.com/site/about/guidelines.xhtml

Tables

Table 1 Comparison of included and excluded participants

Characteristics	Included (N=449,943)	Excluded (N=52,550)
Sex		
Female	240,976 (53.6%)	32,402 (61.7%)
Male	208,967 (46.4%)	20,148 (38.3%)
Average age (SD)	56.5 (8.1)	57.0 (7.8)
BMI[1]		
Underweight	2,307 (0.5%)	321 (0.6%)
Healthy weight	145,082 (32.3%)	15,222 (29.7%)
Overweight	190,177 (42.4%)	22,053 (43.1%)
Obese	110,541 (24.7%)	13,600 (26.6%)
Central obesity[1]		
Not obese	177,869 (39.6%)	17,719 (34.5%)
Central obesity	271,126 (60.4%)	33,642 (65.5%)
Ethnic background		

For peer review only

For peer review only - http://bmjopen.bmj.com/site/about/guidelines.xhtml

White	424,213 (94.4%)	47,901 (91.3%)
Mixed	2,593 (0.6%)	316 (0.6%)
Asian	8,016 (1.8%)	1,823 (3.5%)
Black	7,050 (1.6%)	984 (1.9%)
Chinese	1,407 (0.3%)	167 (0.3%)
Other ethnicity[2]	6,068 (1.4%)	1,267 (2.4%)
Tobacco smoking		
Non-smoker	402,337 (89.6%)	45,860 (88.3%)
Current smoker	46,793 (10.4%)	6,184 (11.9%)
Alcohol drinking status		
Never drink	35,590 (7.9%)	5,071 (9.8%)
Drink occasionally	101,690 (22.7%)	12,208 (23.5%)
Drink weekly	220,709 (49.2%)	24,028 (46.2%)
Drink daily	90,984 (20.3%)	10,713 (20.6%)
Vitamin and mineral supplementation use		

For peer review only - http://bmjopen.bmj.com/site/about/guidelines.xhtml

Vitamin D and associated mineral supplement[3]	285,524 (63.6%)	31,097 (63.9%)
Other vitamin and mineral supplement[4]	163,214 (36.4%)	17,537 (36.1%)
Mean outdoors time in summer (SD)	3.8 (2.4)	3.8 (2.4)
Mean outdoors time in winter (SD)	1.9 (1.8)	1.9 (1.8)
Index of multiple deprivation index (IMD)[5]		
1 (Least deprived)	88,516 (20.2%)	9,616 (18.7%)
2	88,210 (20.1%)	9,782 (19.1%)
3	87,584 (20.0%)	10,249 (20.0%)
4	87,585 (20%)	10,306 (21.0%)
5 (Most deprived)	86,539 (19.7%)	11,370 (22.2%)

1. The classification is suggested by NICE guidelines; 2. Includes any other unlisted ethnic groups, unclear ethnic groups and participants prefer not to answer; 3. Vitamin D and associated mineral: vitamin D, multivitamin, fish oil, and calcium supplementation; 4. Other vitamin and mineral supplements: vitamin A, B, C, E, folic acid or folate, glucosamine, zinc, iron, selenium and other supplements; 5. IMD scores were by quintile.

For peer review only

Table 2 Basic characteristics of the study population

Characteristics	Vitamin D Deficiency (< 25 nmol/L)	Vitamin D Insufficiency (25 – 50 nmol/L)	Vitamin D Sufficiency (> 50 nmol/L)
Serum vitamin D levels (SD) (nmol/L)	19.2 (3.9)	37.8 (7.1)	67.7 (14.5)
Sex			
Female (N=240,263)	32,180 (13.4%)	100,423 (41.8%)	107,660 (44.8%)
Male (N=208,338)	28,507 (13.7%)	87,368 (41.9%)	92,463 (44.4%)
Age (SD)	54.8 (8.2)	56.2 (8.1)	57.3 (8.0)
BMI[1]			
Underweight (N=2,301)	423 (18.3%)	844 (36.7%)	1,034 (45%)
Healthy weight (N=144,591)	15,886 (11.0%)	54,115 (37.4%)	74,590 (51.6%)
Overweight (N=189,583)	22,841 (12.1%)	79,351 (41.9%)	87,391 (46.1%)
Obese (N=110,292)	20,990 (19.0%)	52,704 (47.8%)	36,598 (33.2%)
Central obesity[1]			

For peer review only - http://bmjopen.bmj.com/site/about/guidelines.xhtml

Non-obese (N=177,348)	19,514 (11.0%)	66,998 (37.8%)	90,836 (51.2%)
Central obesity (N=270,306)	40,903 (15.1%)	120,389 (44.5%)	109,014 (40.3%)
Ethnic background			
White (N=422,907)	50,920 (12.0%)	176,195 (41.7%)	195,792 (46.3%)
Mixed (N=2,589)	642 (24.8%)	1,241 (47.9%)	706 (27.3%)
Asian (N=8,000)	4,297 (53.7%)	2,979 (37.3 %)	724 (9.1%)
Black (N=7,046)	2,459 (34.9%)	3,503 (49.7%)	1,084 (15.4%)
Chinese (N=1,405)	381 (27.1%)	787 (56.0%)	237 (16.9%)
Other ethnicity[2] (N=6,059)	1,860 (30.7%)	2,816 (46.5%)	1,383 (22.8%)
Tobacco smoking			
Non-smoker (N=401,069)	50,270 (12.5%)	167,496 (41.8%)	183,303 (45.7%)
Current smoker (N=46,721)	10,170 (21.7%)	19,944 (42.7%)	16,607 (35.6%)
Alcohol drinking status			
Never drink (N=35,503)	8,419 (23.7%)	15,265 (43.0%)	11,819 (33.3%)
Drink occasionally (N=101,398)	16,655 (16.4%)	45,208 (44.6%)	39,535 (39.0%)
Drink weekly (N=219,988)	24,577 (11.1%)	90,836 (41.3%)	104,575 (47.6%)

For peer review only - http://bmjopen.bmj.com/site/about/guidelines.xhtml

For peer review only

Drink daily (N=90,742)	10,730 (11.8%)	36,063 (39.7%)	43,949 (48.4%)
Mean outdoors time in summer (hours) (SD)	3.3 (2.4)	3.6 (2.4)	4.1 (2.4)
Mean outdoors time in winter (hours) (SD)	1.7 (1.8)	1.9 (1.8)	2.1 (1.8)
Vitamin and mineral supplementation use			
Other vitamin or mineral supplement[3] (N=284,768)	48,877 (17.1%)	128,478 (45.1%)	107,413 (37.7%)
Vitamin D and associated mineral[4] supplement (N=162,629)	11,368 (7.0%)	58,801 (36.2%)	92,460 (56.9%)
Index of multiple deprivation (IMD)[5]			
1 (Least deprived, N=88,264)	8,414 (9.5%)	34,962 (39.6%)	44,888 (50.9%)
2 (N=87,909)	9,321 (10.6%)	36,073 (41.0%)	42,515 (48.4%)
3 (N=87,268)	10,480 (12.0%)	36,049 (41.3%)	40,739 (46.7%)
4 (N=87,321)	13,211 (15.1%)	37,646 (43.1%)	36,464 (41.8%)
5 (Most deprived N=86,347)	17,602 (20.4%)	38,369 (44.4%)	30,376 (35.2%)
Seasons			
Spring (N= 129,570)	25,912 (20%)	62,378 (48.1%)	41,280 (31.7%)

For peer review only - http://bmjopen.bmj.com/site/about/guidelines.xhtml

For peer review only

Summer (N= 118,924)	5,385 (4.5%)	39,856 (33.5%)	73,683 (62.0%)
Autumn (N= 108,888)	8,324 (7.6%)	41,496 (38.1%)	59,068 (54.3%)
Winter (N= 91,219)	21,066 (23.1%)	44,061 (48.3%)	26,092 (28.6%)
Regions of UK Biobank assessment centres			
South West (N= 38,872)	3,068 (7.9%)	14,622 (37.6%)	21,182 (54.5%)
South East (N= 39,814)	3,245 (8.2%)	15,400 (38.7%)	21,169 (53.2%)
London (N= 61,291)	10,232 (16.7%)	27,037 (44.1%)	24,022 (39.2%)
East Midlands (N= 30,337)	3,001 (9.9%)	12,115 (39.9%)	15,221 (50.2%)
West Midlands (N= 40,044)	6,785 (17.0%)	17,670 (44.1%)	15,589 (38.9%)
Wales (N= 19,142)	2,732 (14.3%)	8,808 (46.0%)	7,614 (39.8%)
Yorkshire and The Humber (N= 66,197)	8,372 (12.7%)	27,878 (42.1%)	29,947 (45.2%)
North West (N= 68,196)	8,715 (12.8%)	27,924 (41.0%)	31,557 (46.3%)
North East (N=52,277)	6,919 (13.2%)	21,174(40.5%)	24,184 (46.3%)
Scotland (N=32,419)	7,618 (23.5%)	15,163 (46.8%)	9,638 (29.7%)

1. The classification is suggested by NICE guidelines; 2. Includes any other unlisted ethnic groups, unclear ethnic groups and participants prefer not to answer; 3. Vitamin D and associated mineral: vitamin D, multivitamin, fish oil, and calcium supplementation; 4. Other vitamin and mineral supplements: vitamin A, B, C, E, folic acid or folate, glucosamine, zinc, iron, selenium and other supplements; 5. IMD scores were by quintile.

For peer review only - http://bmjopen.bmj.com/site/about/guidelines.xhtml

Table 3 The association between demographic characteristics and low vitamin D status

Characteristics	Odds ratio of vitamin D insufficiency (25(OH)D < 50 nmol/L)		Odds ratio of vitamin D deficiency (25(OH)D < 25 nmol/L)	
	Crude	Adjusted[1]	Crude	Adjusted[1]
Sex				
Female	1	1	1	1
Male	1.02 (1.0, 1.03)	0.91 (0.90, 0.93)	1.02 (1.01, 1.04)	0.91 (0.9, 0.93)
Age (SD)	0.98 (0.97, 0.97)	0.98 (0.98, 0.98)	0.97 (0.96, 0.97)	0.98 (0.98, 0.98)
BMI				
Healthy weight	1	1	1	1
Underweight	1.3 (1.20, 1.42)	1.26 (1.14, 1.38)	1.83 (1.64, 2.03)	1.71 (1.51, 1.93)
Overweight	1.25 (1.23, 1.26)	1.24 (1.22, 1.25)	1.11 (1.09, 1.13)	1.04 (1.01, 1.06)
Obese	2.15 (2.11, 2.18)	2.08 (2.04, 2.12)	1.91 (1.86, 1.95)	1.68 (1.64, 1.72)
Ethnic background				
White	1	1	1	1
Mixed	2.30 (2.11, 2.51)	2.24 (2.03, 2.46)	2.42 (2.21. 2.64)	2.31 (2.09, 2.56)

For peer review only

For peer review only - http://bmjopen.bmj.com/site/about/guidelines.xhtml

Asian	8.67 (8.02, 9.35)	8.54 (7.87, 9.27)	8.47 (8.10,8.86)	10.99 (10.39, 11.62)
Black	4.74 (4.44, 5.06)	4.14 (3.85, 4.45)	3.93 (3.74, 4.13)	3.6 (3.39, 3.83)
Chinese	4.25 (3.70, 4.89)	4.42 (3.81, 5.14)	2.72 (2.42, 3.06)	2.77 (2.42, 3.18)
Other ethnicity	2.91 (2.74, 3.10)	2.73 (2.55, 2.93)	3.24 (3.07, 3.42)	3.11 (2.9, 3.33)
Tobacco smoking				
Non-smoker	1	1	1	1
Current smoker	1.53 (1.50, 1.56)	1.43 (1.40, 1.46)	1.94 (1.90, 1.99)	1.82 (1.77, 1.87)
Alcohol drinking status				
Never drink	1	1	1	1
Drink occasionally	0.78 (0.76, 0.8)	0.85 (0.84, 0.88)	0.63 (0.61, 0.65)	0.75 (0.73, 0.78)
Drink weekly	0.55 (0.53, 0.56)	0.66 (0.64, 0.68)	0.40 (0.39, 0.42)	0.55 (0.53, 0.57)
Drink daily	0.53 (0.52, 0.55)	0.70 (0.68, 0.72)	0.43 (0.42, 0.45)	0.66 (0.64, 0.69)
Vitamin and mineral supplementation				
Other vitamin and mineral supplement	1	1	1	1

For peer review only

For peer review only - http://bmjopen.bmj.com/site/about/guidelines.xhtml

Vitamin D, multivitamin, fish oil and calcium supplement	0.46 (0.45, 0.47)	0.41 (0.41, 0.42)	0.36 (0.35, 0.37)	0.32 (0.31, 0.33)
Index of multiple deprivation (IMD)				
1 (Least deprived)	1	1	1	1
2	1.11 (1.09, 1.13)	1.03 (1.01, 1.05)	1.12 (1.09,1.16)	1.02 (0.98, 1.05)
3	1.18 (1.16, 1.2)	1.05 (1.03,1.07)	1.29 (1.26,1.33)	1.11 (1.07, 1.14)
4	1.44 (1.42, 1.47)	1.17 (1.14,1.19)	1.69 (1.64,1.74)	1.27 (1.23, 1.31)
5 (Most deprived)	1.91 (1.87, 1.94)	1.34 (1.31,1.37)	2.43 (2.36, 2.50)	1.53 (1.48, 1.58)
Seasons				
Summer	1	1	1	1
Spring	3.48 (3.42, 3.54)	3.86 (3.79, 3.93)	5.26 (5.1, 5.42)	6.43 (6.22, 6.65)
Autumn	1.37 (1.35, 1.40)	1.43 (1.40, 1.45)	1.74 (1.68, 1.80)	1.89 (1.82, 1.96)
Winter	4.06 (3.99, 4.14)	4.56 (4.47, 4.65)	6.31 (6.12, 6.51)	7.82 (7.55, 8.1)
Regions (categorized centres)				
South West	1	1	1	1
South East	1.05 (1.03, 1.08)	1.15 (1.11, 1.18)	1.04 (0.98, 1.09)	1.11 (1.05, 1.18)

For peer review only - http://bmjopen.bmj.com/site/about/guidelines.xhtml

London	1.86 (1.81, 1.91)	1.31 (1.28, 1.35)	2.34 (2.24, 2.44)	1.31 (1.25, 1.38)
East Midlands	1.19 (1.15, 1.23)	1.07 (1.04, 1.11)	1.28 (1.22, 1.35)	1.13 (1.07, 1.2)
West Midlands	1.88 (1.82, 1.93)	1.22 (1.18, 1.26)	2.38 (2.27, 2.48)	1.26 (1.19, 1.32)
Wales	1.81 (1.75, 1.88)	1.03 (0.99, 1.07)	1.94 (1.84, 2.05)	1.03 (0.97, 1.09)
Yorkshire and The Humber	1.45 (1.41, 1.48)	1.19(1.16, 1.23)	1.68 (1.61, 1.76)	1.30 (1.24, 1.36)
North West	1.38 (1.35, 1.42)	1.15 (1.12, 1.18)	1.68 (1.61, 1.76)	1.28 (1.22, 1.34)
North East	1.39 (1.35, 1.43)	1.23 (1.19, 1.27)	1.78 (1.7, 1.86)	1.53 (1.46, 1.61)
Scotland	2.83 (2.74, 2.92)	1.98 (1.91, 2.05)	3.58 (3.43, 3.75)	2.39 (2.28, 2.51)

321 1. Adjusted for sex, age, BMI categories, ethnicity, smoking, drinking, vitamin D supplementation, index of multiple deprivation,
322 seasons, regions of UK Biobank assessment centres; 3. IMD scores were by quintile.

For peer review only

Authors' contribution

LYL contributed to the design of the study, analysed the data, drafted and revised the manuscript according to other authors' comments; CWG contributed to the design of the study, made critical comments on the manuscript and revised the paper critically; SML contributed to the design of the study, made critical comments on the manuscript and revised the paper critically; LS contributed to the design of the study and revised the paper critically. All authors approved the final version of the protocol.

Competing interest:

None declared.

Funding statement:

LYL is funded by the scholarship of government sponsorship for overseas study by the Ministry of Education Republic of China (Taiwan). CWG is supported by a Wellcome Intermediate Clinical Fellowship (201440_Z_16_Z). SML is funded by a Wellcome Senior Clinical Fellowship in Science (205039/Z/16/Z).

Data sharing statement:

Other researchers can apply for UK Biobank data to answer specific research questions. We will upload our analysis codes to LSHTM Data Compass, an open data repository for research outputs from LSHTM (https://datacompass.lshtm.ac.uk/).

Acknowledgement

We thank Yi-Tin Ching for assistance with technical support of ArcGIS mapping.

Patient consent for publication:

Not required

For peer review only

References:

1. Holick MF. Vitamin D deficiency. *The New England journal of medicine* 2007;357(3):266-81. doi: 10.1056/NEJMra070553 [published Online First: 2007/07/20]
2. Public Health England. PHE publishes new advice on vitamin D 2016 [Available from: https://www.gov.uk/government/news/phe-publishes-new-advice-on-vitamin-d accessed 05 October 2020.
3. Sassi F, Tamone C, D'Amelio P. Vitamin D: Nutrient, Hormone, and Immunomodulator. *Nutrients* 2018;10(11) doi: 10.3390/nu10111656 [published Online First: 2018/11/08]
4. Mann EH, Pfeffer PE, Hawrylowicz CM. Chapter 104 - Vitamin D and Adaptive Immunology in Health and Disease. In: Feldman D, ed. Vitamin D (Fourth Edition): Academic Press 2018:937-49.
5. Martineau AR, Jolliffe DA, Hooper RL, et al. Vitamin D supplementation to prevent acute respiratory tract infections: systematic review and meta-analysis of individual participant data. *Bmj* 2017;356:i6583. doi: https://dx.doi.org/10.1136/bmj.i6583
6. The Scientific Advisory Committee on Nutrition. Vitamin D and Health: Public Health England 2016.
7. Royal Osteoporosis Society. Vitamin D and Bone Health: A Practical Clinical Guideline for Patient Management. 2 ed: Royal Osteoporosis Society,, 2018:10.
8. Hypponen E, Power C. Hypovitaminosis D in British adults at age 45 y: nationwide cohort study of dietary and lifestyle predictors. *The American journal of clinical nutrition* 2007;85(3):860-8. doi: 10.1093/ajcn/85.3.860 [published Online First: 2007/03/09]
9. Public Health England. National Diet and Nutrition Survey Results from Years 1, 2, 3 and 4 (combined) of the Rolling Programme (2008/2009 – 2011/2012). In: Bates B, Lennox A, Prentice A, et al., eds. National Diet and Nutrition Survey (NDNS): Public Health England,, 2017.
10. Sudlow C, Gallacher J, Allen N, et al. UK biobank: an open access resource for identifying the causes of a wide range of complex diseases of middle and old age. *PLoS Med* 2015;12(3):e1001779. doi: 10.1371/journal.pmed.1001779 [published Online First: 2015/04/01]
11. UK Biobank. UK Biobank Biomarker Panel, 2015.
12. Fry A, Littlejohns TJ, Sudlow C, et al. Comparison of Sociodemographic and Health-Related Characteristics of UK Biobank Participants With Those of the General Population. *American journal of epidemiology* 2017;186(9):1026-34. doi: https://dx.doi.org/10.1093/aje/kwx246
13. UK Biobank. Index of Multiple Deprivation Scores 2018.
14. Elliott P, Peakman TC, Biobank UK. The UK Biobank sample handling and storage protocol for the collection, processing and archiving of human blood and urine. *International journal of epidemiology* 2008;37(2):234-44. doi: 10.1093/ije/dym276 [published Online First: 2008/04/03]
15. CDC. CDC Vitamin D Standardization-Certification Program (CDC VDSCP), 2020.
16. UK Biobank. Biomarker assay quality procedures: approaches used to minimise systematic and random errors. 1.2 ed, 2019.
17. UK Biobank. Companion document for serum biomarker data. 1 ed, 2019.
18. Pearce SH, Cheetham TD. Diagnosis and management of vitamin D deficiency. *Bmj* 2010;340:b5664. doi: 10.1136/bmj.b5664
19. National Institute for Health and Care Excellence (NICE). BMI: preventing ill health and premature death in black, Asian and other minority ethnic groups: National Institute for Health and Clinical Excellence 2013.
20. Office for National Statistics. Regions (December 2017) Map in United Kingdom 2019 [Available from: https://geoportal.statistics.gov.uk/datasets/0758ee9479044ecc9547ebab340c84a8 accessed 09/23 2020.
21. Met Office. Seasons 2019 [Available from: https://www.metoffice.gov.uk/weather/learn-about/weather/seasons accessed 25/11 2019.
22. World Medical A. World Medical Association Declaration of Helsinki: ethical principles for medical research involving human subjects. *JAMA* 2013;310(20):2191-94. doi: 10.1001/jama.2013.281053
23. Darling AL, Hart KH, Macdonald HM, et al. Vitamin D deficiency in UK South Asian Women of childbearing age: a comparative longitudinal investigation with UK Caucasian women. *Osteoporos Int* 2013;24(2):477-88. doi: 10.1007/s00198-012-1973-2 [published Online First: 2012/04/25]

For peer review only

24. Renzaho AMN, Halliday JA, Nowson C. Vitamin D, obesity, and obesity-related chronic disease among ethnic minorities: A systematic review. *Nutrition* 2011;27(9):868-79. doi: https://doi.org/10.1016/j.nut.2010.12.014
25. Santos A, Amaral TF, Guerra RS, et al. Vitamin D status and associated factors among Portuguese older adults: results from the Nutrition UP 65 cross-sectional study. *BMJ open* 2017;7(6):e016123. doi: 10.1136/bmjopen-2017-016123 [published Online First: 2017/06/25]
26. Liu X, Baylin A, Levy PD. Vitamin D deficiency and insufficiency among US adults: prevalence, predictors and clinical implications. *Br J Nutr* 2018;119(8):928-36. doi: 10.1017/s0007114518000491 [published Online First: 2018/04/13]
27. Tardelli VS, Lago M, Silveira DXD, et al. Vitamin D and alcohol: A review of the current literature. *Psychiatry Res* 2017;248:83-86. doi: 10.1016/j.psychres.2016.10.051 [published Online First: 2016/12/30]

For peer review only

Supplementary figures

Supplementary figure 1 Mean percentage of participants with vitamin D deficiency by UK Biobank assessment centres and season

For peer review only

19

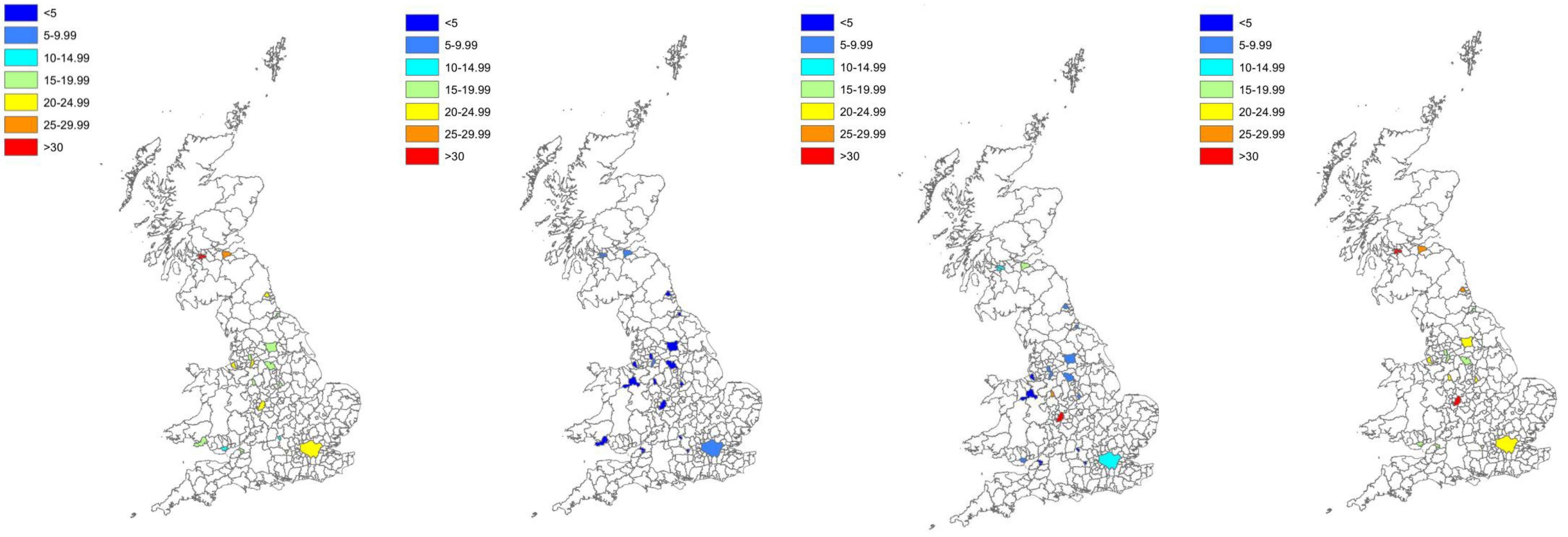

a. Spring b. Summer c. Autumn d. Winter

STROBE 2007 (v4) checklist of items to be included in reports of observational studies in epidemiology*

Checklist for cohort, case-control, and cross-sectional studies (combined)

Section/Topic	Item #	Recommendation	Reported on page #
**Title and abstract**	1	(*a*) Indicate the study's design with a commonly used term in the title or the abstract	#1
		(*b*) Provide in the abstract an informative and balanced summary of what was done and what was found	#4
**Introduction**			
Background/rationale	2	Explain the scientific background and rationale for the investigation being reported	#6
Objectives	3	State specific objectives, including any pre-specified hypotheses	#6
**Methods**			
Study design	4	Present key elements of study design early in the paper	#8
Setting	5	Describe the setting, locations, and relevant dates, including periods of recruitment, exposure, follow-up, and data collection	#7
Participants	6	(*a*) *Cohort study*—Give the eligibility criteria, and the sources and methods of selection of participants. Describe methods of follow-up<br>*Case-control study*—Give the eligibility criteria, and the sources and methods of case ascertainment and control selection. Give the rationale for the choice of cases and controls<br>*Cross-sectional study*—Give the eligibility criteria, and the sources and methods of selection of participants	#7
		(*b*) *Cohort study*—For matched studies, give matching criteria and number of exposed and unexposed<br>*Case-control study*—For matched studies, give matching criteria and the number of controls per case	Not applicable
Variables	7	Clearly define all outcomes, exposures, predictors, potential confounders, and effect modifiers. Give diagnostic criteria, if applicable	#7
Data sources/ measurement	8*	For each variable of interest, give sources of data and details of methods of assessment (measurement). Describe comparability of assessment methods if there is more than one group	#7
Bias	9	Describe any efforts to address potential sources of bias	#8
Study size	10	Explain how the study size was arrived at	#7

For peer review only

Quantitative variables	11	Explain how quantitative variables were handled in the analyses. If applicable, describe which groupings were chosen and why	#7
Statistical methods	12	(*a*) Describe all statistical methods, including those used to control for confounding	#8
		(*b*) Describe any methods used to examine subgroups and interactions	Not applicable
		(*c*) Explain how missing data were addressed	Not applicable
		(*d*) *Cohort study*—If applicable, explain how loss to follow-up was addressed<br>*Case-control study*—If applicable, explain how matching of cases and controls was addressed<br>*Cross-sectional study*—If applicable, describe analytical methods taking account of sampling strategy	Not applicable
		(*e*) Describe any sensitivity analyses	Not applicable
**Results**			
Participants	13*	(a) Report numbers of individuals at each stage of study—eg numbers potentially eligible, examined for eligibility, confirmed eligible, included in the study, completing follow-up, and analysed	#9
		(b) Give reasons for non-participation at each stage	#9
		(c) Consider use of a flow diagram	
Descriptive data	14*	(a) Give characteristics of study participants (eg demographic, clinical, social) and information on exposures and potential confounders	#10
		(b) Indicate number of participants with missing data for each variable of interest	#13
		(c) *Cohort study*—Summarise follow-up time (eg, average and total amount)	Not applicable
Outcome data	15*	*Cohort study*—Report numbers of outcome events or summary measures over time	Not applicable
		Case-control study—Report numbers in each exposure category, or summary measures of exposure	Not applicable
		Cross-sectional study—Report numbers of outcome events or summary measures	#10, #15
Main results	16	(*a*) Give unadjusted estimates and, if applicable, confounder-adjusted estimates and their precision (eg, 95% confidence interval). Make clear which confounders were adjusted for and why they were included	Not applicable
		(*b*) Report category boundaries when continuous variables were categorized	#15

For peer review only

		(*c*) If relevant, consider translating estimates of relative risk into absolute risk for a meaningful time period	Not applicable
Other analyses	17	Report other analyses done—eg analyses of subgroups and interactions, and sensitivity analyses	Not applicable
**Discussion**			
Key results	18	Summarise key results with reference to study objectives	#10
Limitations	19	Discuss limitations of the study, taking into account sources of potential bias or imprecision. Discuss both direction and magnitude of any potential bias	#11
Interpretation	20	Give a cautious overall interpretation of results considering objectives, limitations, multiplicity of analyses, results from similar studies, and other relevant evidence	#11
Generalisability	21	Discuss the generalisability (external validity) of the study results	#12
**Other information**			
Funding	22	Give the source of funding and the role of the funders for the present study and, if applicable, for the original study on which the present article is based	#2

*Give information separately for cases and controls in case-control studies and, if applicable, for exposed and unexposed groups in cohort and cross-sectional studies.

Note: An Explanation and Elaboration article discusses each checklist item and gives methodological background and published examples of transparent reporting. The STROBE checklist is best used in conjunction with this article (freely available on the Web sites of PLoS Medicine at http://www.plosmedicine.org/, Annals of Internal Medicine at http://www.annals.org/, and Epidemiology at http://www.epidem.com/). Information on the STROBE Initiative is available at www.strobe-statement.org.

For peer review only

BMJ Open

The distribution of Vitamin D status in the UK: a cross-sectional analysis of UK Biobank

Journal:	*BMJ Open*
Manuscript ID	bmjopen-2020-038503.R2
Article Type:	Original research
Date Submitted by the Author:	18-Dec-2020
Complete List of Authors:	Lin, Liang-Yu; London School of Hygiene and Tropical Medicine Faculty of Epidemiology and Population Health, Department of Non-communicable Disease Epidemiology<br>Smeeth, Liam; London School of Hygiene and Tropical Medicine,<br>Langan, Sinead; London School of Hygiene and Tropical Medicine Faculty of Epidemiology and Population Health, Department of Non-communicable Disease Epidemiology<br>Warren-Gash, Charlotte; London School of Hygiene and Tropical Medicine Faculty of Epidemiology and Population Health, Department of Non-Communicable Disease Epidemiology
**Primary Subject Heading**:	Epidemiology
Secondary Subject Heading:	Nutrition and metabolism
Keywords:	EPIDEMIOLOGY, NUTRITION & DIETETICS, PUBLIC HEALTH

SCHOLARONE™
Manuscripts

I, the Submitting Author has the right to grant and does grant on behalf of all authors of the Work (as defined in the below author licence), an exclusive licence and/or a non-exclusive licence for contributions from authors who are: i) UK Crown employees; ii) where BMJ has agreed a CC-BY licence shall apply, and/or iii) in accordance with the terms applicable for US Federal Government officers or employees acting as part of their official duties; on a worldwide, perpetual, irrevocable, royalty-free basis to BMJ Publishing Group Ltd ("BMJ") its licensees and where the relevant Journal is co-owned by BMJ to the co-owners of the Journal, to publish the Work in this journal and any other BMJ products and to exploit all rights, as set out in our licence.

The Submitting Author accepts and understands that any supply made under these terms is made by BMJ to the Submitting Author unless you are acting as an employee on behalf of your employer or a postgraduate student of an affiliated institution which is paying any applicable article publishing charge ("APC") for Open Access articles. Where the Submitting Author wishes to make the Work available on an Open Access basis (and intends to pay the relevant APC), the terms of reuse of such Open Access shall be governed by a Creative Commons licence – details of these licences and which Creative Commons licence will apply to this Work are set out in our licence referred to above.

Other than as permitted in any relevant BMJ Author's Self Archiving Policies, I confirm this Work has not been accepted for publication elsewhere, is not being considered for publication elsewhere and does not duplicate material already published. I confirm all authors consent to publication of this Work and authorise the granting of this licence.

For peer review only

Manuscript title:

The distribution of Vitamin D status in the UK: a cross-sectional analysis of UK Biobank

Authors' full names:

Liang-Yu Lin[1]. LSHTM liang-yu.lin@lshtm.ac.uk

ORCID: https://orcid.org/0000-0003-4720-6738

Liam Smeeth[1]. LSHTM Liam.Smeeth@lshtm.ac.uk

ORCID: https://orcid.org/0000-0002-9168-6022

Sinéad M Langan[1]*. LSHTM Sinead.Langan@lshtm.ac.uk

ORCID: https://orcid.org/0000-0002-7022-7441

Charlotte Warren-Gash[1]*. LSHTM Charlotte.Warren-Gash1@lshtm.ac.uk

ORCID: https://orcid.org/0000-0003-4524-3180

* The senior authors contributed equally to this work

Authors' Affiliations:

1. Faculty of Epidemiology & Population Health, London School of Hygiene and Tropical Medicine, London, UK

# Correspondence to:

Dr Liang-Yu Lin, LSHTM, Keppel Street, London, WC1E 7HT, United Kingdom

Tel: +44 (0) 20 7636 8636 liang-yu.lin@lshtm.ac.uk

For peer review only

20 Keywords:

21 Vitamin D, Vitamin D deficiency, Seasons, Geographic locations, Ethnicity

22 Word count: 2808

For peer review only

For peer review only - http://bmjopen.bmj.com/site/about/guidelines.xhtml

ABSTRACT

Objective

No recent large studies have described the distribution of vitamin D status in the UK. Understanding the epidemiology of vitamin D deficiency is important to inform targeted public health recommendations. This study aimed to investigate the distribution of factors associated with serum vitamin D status in a large national cohort.

Design

A cross-sectional study.

Setting

The UK Biobank, a prospective cohort study following the health and wellbeing of middle-aged and older adults recruited between 2006 and 2010.

Participants

A total of 449,943 participants aged 40 to 69 years with measured serum vitamin D status were eligible for the analysis. Participants completed a questionnaire about sex, age, ethnic background, vitamin D supplementation, smoking, drinking and socioeconomic status.

Primary and secondary outcome measures

We investigated the distribution of serum vitamin D status and the association between demographic factors and vitamin D deficiency or insufficiency. Vitamin D deficiency was defined as a serum 25-hydroxyvitamin D level <25 nmol/L.

For peer review only

Multivariable logistic regression was used to assess the association between demographic factors and vitamin D status.

Results

Asian (n=4,297/8000, 53.7%) and Black (n=2,459/7046, 34.9%) participants had a higher proportion of vitamin D deficiency than white participants (n=50,920/422,907, 12%). During spring and winter, the proportion of vitamin D deficiency was higher across the UK and higher in the north than in the south. Male sex, abnormal body mass index, non-white ethnic backgrounds, smoking, and being more socioeconomically deprived were associated with higher odds of vitamin D deficiency.Increasing age, taking vitamin D supplements and drinking alcohol were associated with lower odds of deficiency.

Conclusions

Vitamin D status varied among different ethnic groups and by season and geographical area within the UK. Taking supplements was associated with a lower risk of vitamin D deficiency. These findings support the vitamin D supplementation recommendations of Public Health England.

Abstract words count: 299

For peer review only

For peer review only - http://bmjopen.bmj.com/site/about/guidelines.xhtml

Strengths and limitations of this study

- This study used a large cohort of British adults in middle and older age, and its large sample size provides more statistical power in assessing associations.
- The findings provide further evidence to support the nutritional supplementation recommendations of Public Health England.
- The cohort is not nationally representative, so the findings cannot be extrapolated to the general population.
- The questionnaire on vitamin supplementation was self-reported and thus cannot be used to estimate the required supplement amount.

For peer review only

For peer review only - http://bmjopen.bmj.com/site/about/guidelines.xhtml

INTRODUCTION

Vitamin D is essential to bone formation. It is mainly produced by the skin after sun exposure, and it can also be obtained from food and supplementation. After production or ingestion, it is further metabolised in the liver into 25-hydroxyvitamin D, which can be measured in the blood. 25-hydroxyvitamin D is further transformed in the kidneys into 1,25-dihydroxyvitamin D, the active form of vitamin D. This active vitamin D metabolite acts on the intestines and kidneys, regulates the absorption and excretion of calcium and phosphate, and facilitates the mineralisation of bone. Vitamin D deficiency may impair bone mineralisation, leading to osteopenia or osteoporosis[1]. Currently, Public Health England suggests that people older than 4 years of age should take 10 μg (400 IU) of vitamin D daily as a supplement during the winter to support musculoskeletal health[2].

In addition to the classical effects of mineral homeostasis, more recent attention has focused on novel effects of vitamin D. Previous studies have indicated that vitamin D may have potential immunomodulatory effects. Active vitamin D can enhance innate immunity by increasing the production of antimicrobial peptides, and vitamin D can regulate adaptive immunity as well[3]. Epidemiological studies have also indicated that vitamin D is associated with autoimmune diseases such as inflammatory bowel disease, type I diabetes mellitus and multiple sclerosis[4]. In addition, a meta-analysis using original data from trials suggested that taking vitamin D supplementation may decrease the risk of respiratory infections[5].

Although 1,25-dihydroxyvitamin D plays an active role in metabolism, its half-life is less than 4 hours, while the half-life of 25-hydroxyvitamin D is around 2–3 weeks. Thus, clinical 25-hydroxyvitamin D levels in the blood have been used to assess vitamin D status[6]. 25-hydroxyvitamin D can be analysed using either

For peer review only - http://bmjopen.bmj.com/site/about/guidelines.xhtml

chemiluminescence immunoassay or tandem mass spectrometry, which are recognised by the Royal Osteoporosis Society and Public Health England[6 7]. However, no current consensus exists about the definition of vitamin D deficiency, so each study may use different standards. The Endocrine Society of the US defined vitamin D deficiency as 25-hydroxyvitamin D below 50 nmol/L, while the criterion of Public Health England is 25-hydroxyvitamin D less than 25 nmol/L.

Using the criterion of a blood vitamin D level less than 25 nmol/L, a cohort of British Caucasians (n=7,437) indicated that in winter and spring, the average prevalence of vitamin D deficiency was 15.5% among people who were 45 years old[8]. Similarly, among adults in the National Diet and Nutrition Survey in the UK (n=3,450), the prevalence of vitamin D deficiency was approximately 24.0% in men and 21.7% in women aged between 19 and 64 years[9]. However, these studies were relatively small, and no recent large study has described the distribution of vitamin D status in the UK.

The UK Biobank is a prospective, nationwide cohort used to investigate risk factors for major diseases in middle and old age; cohort participants were assessed for various biochemical biomarkers, including serum vitamin D status[10 11]. By using UK Biobank data, we aimed to conduct a cross-sectional study to investigate different demographic, seasonal and regional factors associated with vitamin D deficiency distribution in the UK.

METHODS

Study population

The UK Biobank was compiled from 2006 to 2010 by recruiting participants throughout the UK. People aged 40 to 69 years who lived within 40 km of one of the

For peer review only - http://bmjopen.bmj.com/site/about/guidelines.xhtml

22 UK Biobank assessment centres and who were registered with the UK National Health Service were eligible to be included in the cohort; approximately 500,000 volunteers were recruited[10 12]. UK Biobank participants received a wide range of examinations, including questionnaires and physical measures, and blood, urine and saliva samples were assayed[6]. Only participants with results for serum vitamin D status were included in our analyses.

Measurement of covariates

At each participant's first visit to a UK Biobank assessment centre, a touchscreen questionnaire collected basic demographic characteristics; sociodemographic, environmental and lifestyle factors; and the date of assessment[10]. Information such as sex, age, ethnic background, skin colour, smoking status, drinking status, sun exposure, and vitamin and mineral supplement use were recorded. Physical measurements, including body mass index (BMI) and waist circumference were also taken. The index of multiple deprivation (IMD) of participants was obtained through data linkage[13].

Defining serum vitamin D status

Biochemical assays were performed on blood samples collected during the baseline evaluation at the assessment centres [6]. Briefly, serum samples were collected in a silica clot accelerator tube and stored at -80°C. These samples were later processed in a central laboratory using an automated dispensing system[14]. Serum 25-hydroxyvitamin D status was measured by chemiluminescence immunoassay (DiaSorin Ltd. LIASON XL, Italy), which was certified by the Vitamin D Standardization-Certification Program of the Centers for Disease Control and Prevention[15]. To ensure the precision of analysis, quality control samples at different

For peer review only

concentrations were analysed[16], and the testing assay for vitamin D was verified through the RIQAS Immunoassay Speciality I EQA programme (Randox Laboratories Ltd.), an external quality assurance scheme[17].

Statistical analysis

This was a cross-sectional study describing the distribution of serum vitamin D status, so only participants who had at least one available measurement of serum vitamin D status were included. We used the standards of vitamin D deficiency adopted by Public Health England[6 18]. A serum 25-hydroxyvitamin D level less than 25 nmol/L was coded as "deficiency", and 25-50 nmol/L was coded as "insufficiency". A 25-hydroxyvitamin D level greater than 50 nmol/L was coded as "sufficiency". Vitamin D was coded as missing in cases of no reportable value, values above or below the reportable limits, or unrecoverable aliquot problems.

To compare the distribution of vitamin D status by these factors associated with vitamin D, continuous covariates, such as BMI and waist circumference, were coded as categorical variables following National Institute for Health and Care Excellence (NICE) guidance[19]. According to the guidelines, different BMI and waist circumference standards were applied for Asian and Chinese populations. Self-reported ethnic backgrounds were coded into six groups according to the original questionnaire. Participants with any other unlisted ethnic group, with an unclear ethnic group or preferring not to answer were assigned "other ethnic group". The mean outdoor time was recorded as 0.5 hours/day if it was less than 1 hour. Responses regarding vitamin and mineral use were further regrouped as "vitamin D, multivitamin, fish oil and calcium use" and "other vitamin and mineral use". IMD scores were categorised into five quintiles, with the fifth quintile assigned the "most deprived" group. The locations of 22 Biobank assessment centres were grouped

For peer review only - http://bmjopen.bmj.com/site/about/guidelines.xhtml

For peer review only

using the geographical regions of the UK[20]. The date of blood collection for vitamin D examination was categorised into four seasons, according to the Met Office definitions[21].

Demographic factors including sex, age (modelled as a continuous variable), ethnicity, BMI, smoking, drinking alcohol, IMD, vitamin D testing season, and geographical location were further analysed in terms of their association with vitamin D insufficiency (<50 nmol/L) and deficiency (<25 nmol/L) using simple and multivariable logistic regression models. Covariates such as central obesity were not included in the model to avoid probable collinearity with BMI. All statistical analysis was carried out using Stata/MP version 15 (StataCorp, USA).

Patient and public involvement

Patients and/or the public were not involved in this retrospective study.

Ethical approval

The UK Biobank obtained ethics approval from its Research Ethics Committee (reference: 11/NW/0382). Our study received ethics approval from the UK Biobank (ID:51265) and the Research Ethics Committee of the London School of Hygiene and Tropical Medicine (reference: 17158). Our study followed the principles of the Declaration of Helsinki[22].

RESULTS

Comparison of included and excluded participants

We compared the included participants with those who did not have their vitamin D status tested (**Table 1**). Vitamin D values were missing for 52,550 participants, who were excluded. Among excluded participants, the proportion of female participants (61.7%) was slightly higher than among the included participants (53.6%), while the

For peer review only

For peer review only - http://bmjopen.bmj.com/site/about/guidelines.xhtml

distribution of the other categorical variables was similar across the included and excluded groups. The average age and mean outdoor time in summer were similar among included and excluded participants, while included participants spent more time outside in winter.

Demographic distribution of vitamin D status

The demographic characteristics according to vitamin D status are shown in **Table 2**. The average vitamin D levels were 19.2 nmol/L in the deficient group, 37.8 nmol/L in the insufficient group and 67.7 nmol/L in the sufficient group. The distribution of vitamin D status was similar in both sexes, and the mean age of the vitamin D sufficiency group was older than other groups. The proportion of vitamin deficiency was lower among people with healthy weight (n=15,886, 11%) and non-obese people (n=177,348, 11%). Asian (n=4,297, 53.7%) and Black (n=2,459, 34.9%) people had a higher proportion of vitamin D deficiency than white participants (n=50,920, 12.0%). Vitamin D deficiency was more prevalent among current smokers (n=10,170, 21.7%) and those who never drank alcohol (n=8,419, 23.7%). People deficient in vitamin D spent less outdoor time in winter (mean = 1.7 hours). The proportion of vitamin D deficiency was lower among participants taking vitamin D and associated mineral supplements (n=11,368, 7%) compared with people taking any other supplements (n= 48,877, 17%). The most deprived people had the highest proportion of vitamin D deficiency (n=17,602, 20.4%), while the least deprived participants had the lowest deficiency proportion (n=8,414, 9.5%).

Vitamin D deficiency was more common in winter (n=21,066, 23.1%) and spring (25,912, 20%). Participants who visited assessment centres in Scotland had the highest proportion of vitamin D deficiency (n=7,618, 23.5%) compared with other assessment centres in the southern UK (**Table 2**). A map showing the distribution of

For peer review only - http://bmjopen.bmj.com/site/about/guidelines.xhtml

the proportion of vitamin D deficiency in different seasons by each UK Biobank assessment centre is illustrated in **Supplementary Figure 1**. This shows that in winter and spring, vitamin D deficiency was prevalent across the country, while in summer and autumn it was less common. Furthermore, in spring, autumn and winter, the proportion of vitamin D deficiency in the northern part of the UK was higher than in the southern part of the country (**Supplementary Figure 1**).

Association between demographic factors and vitamin D status

To assess the association of factors related to vitamin D status, the odds ratio of having vitamin D deficiency (25(OH)D <25 nmol/L) or insufficiency (25(OH)D <50 nmol/L) were summarised in **Table 3**. Male sex, abnormal BMI, smoking, non-white ethnicity, and being more deprived had greater odds of vitamin D deficiency or insufficiency. Increasing age, drinking alcohol, and taking vitamin D, multivitamin, fish oil or calcium supplements were associated with lower odds of vitamin D deficiency or insufficiency. Compared with summer, receiving testing in spring or winter had greater odds of vitamin D deficiency or insufficiency. For the regions with assessment centres, Scotland had higher odds of vitamin D deficiency and insufficiency compared with other regions in the UK. After adjusting for the variables listed in Table 3, these associations remained (**Table 3**).

For peer review only

DISCUSSION

In this cross-sectional study, we found that vitamin D deficiency was more prevalent among people of colour, in spring and winter, and in the northern UK. Male sex, abnormal BMI, non-white ethnic background, smoking and being more deprived increased the odds of vitamin D deficiency. Taking vitamin D supplements, drinking

For peer review only - http://bmjopen.bmj.com/site/about/guidelines.xhtml

alcohol, and increasing age were associated with decreased odds of vitamin D deficiency.

Our study showed that vitamin D status was strongly associated with seasonality and geographical distribution, which is similar to previous studies[8 9]. Other studies have also indicated that vitamin D deficiency is more prevalent among non-white ethnic groups, such as south Asians. A small longitudinal study (n=140) in the UK showed that more than 90% of south Asian women in the cohort had vitamin D insufficiency (25-hydroxyvitamin D <50 nmol/L) throughout the year, while the proportion of insufficiency was much lower in the white comparison groups (20.3% in summer and 70% in winter)[23]. A systematic review of 19 cross-sectional studies and one cohort study indicated that serum hydroxyvitamin D levels were lower among Black, African American and non-Hispanic Black groups, compared with Caucasian groups [24]. These findings indicate the importance of differences in ethnicity, latitude and season, which should always be considered in dietary guidelines and future nutritional studies of vitamin D.

We found that abnormal BMI, tobacco smoking, and deprivation increased the odds of vitamin D deficiency or insufficiency, while taking vitamin D and associated mineral supplements was associated with a lower risk of vitamin D deficiency or insufficiency. These results are consistent with other research about factors associated with vitamin D[1 25 26]. The common vitamin D supplements contain around 400 to 2,000 IU of vitamin D_2 or D_3, meeting the official daily intake guide. Our findings provide some evidence about the effect of vitamin D supplements as well as the Public Health England recommendation for vitamin D supplementation in winter and for some ethnic backgrounds[2].

For peer review only - http://bmjopen.bmj.com/site/about/guidelines.xhtml

Curiously, age and drinking alcohol were inversely associated with vitamin D status. Aging has been regarded as a risk factor for vitamin D deficiency; however, our results showed that increasing age slightly decreased the odds of deficiency. Nevertheless, the mean age of our study population was 56.5 years, and age-related vitamin D deficiency tends to manifest at a more advanced age[27]. Another possible explanation is that our study population focused on middle-aged or older participants, who may be more likely to receive prescriptions containing vitamin D. However, this information was not included in our study. Regarding drinking alcohol, although a previous systematic review reported no consistent correlation between alcohol intake and serum vitamin D levels[28], recent studies have shown a negative association. A study using the National Health and Nutrition Examination Survey (NHANES) database in the US from 2001 to 2010 showed the prevalence of vitamin D deficiency among current alcohol drinkers was 38% lower than in non-drinkers[26]. Similarly, a cross-sectional survey in Portugal among 1,500 participants aged over 65 years showed that alcohol drinkers had lower odds of vitamin D deficiency compared with non-drinkers (moderate drinker: OR=0.49 [CI: 0.32 to 0.73]; excessive drinker: OR=0.48 [CI: 0.27 to 0.85])[25]. The mechanism behind this possible association is still unclear, and future studies about vitamin D should take alcohol into account.

The key strengths of our study are its large sample size and wide range of measurements, which provide more statistical power in analysing the factors associated with vitamin D deficiency. Moreover, information about the exposure and outcomes was collected following a pre-defined protocol, and samples were processed systematically, which minimises potential differential or non-differential misclassification bias. Our analysis about the association between demographic

For peer review only

For peer review only - http://bmjopen.bmj.com/site/about/guidelines.xhtml

factors and vitamin D deficiency is solid. However, several important limitations need to be considered. Despite the large size of the UK Biobank, this cohort is not nationally representative. Compared with non-participants, the participants of the UK Biobank were more likely to be older, female, and with a higher socioeconomic status and fewer health conditions[12]. Due to this healthy volunteer effect, our findings on vitamin D status cannot be generalised to the UK population, and we may have underestimated the prevalence of vitamin D deficiency. Nevertheless, as the healthy volunteer effect does not affect the validity of exposure–outcome relationships, our findings on the factors associated with vitamin D status are likely to be valid.

This analysis has concentrated on vitamin D status using serum vitamin D levels obtained from a single blood test, which may not reflect long-term vitamin D status. Additionally, the questionnaires collecting information about vitamin D supplementation and outdoor physical activity could not precisely quantify the exposure. In further research, a more representative sample should be considered to assess the prevalence of vitamin D deficiency in the UK. Researchers may consider using repeated measurements to assess long-term vitamin D status more precisely or including clinical diagnosis or vitamin D prescription to capture clinical status. In addition, collecting information to quantify vitamin D supplementation and outdoor activities in different seasons can further validate the recommendation of Public Health England.

CONCLUSION

Vitamin D deficiency was more common in winter and spring, and its prevalence was higher in the northern UK than the southern UK. Male sex, abnormal BMI, Asian and Black ethnic backgrounds, and tobacco smoking were associated with higher odds of

For peer review only

For peer review only - http://bmjopen.bmj.com/site/about/guidelines.xhtml

vitamin D deficiency. Taking vitamin D supplements and drinking alcohol were associated with lower odds of vitamin D deficiency. These results provide some evidence supporting the Public Health England recommendation for taking vitamin D supplementation in winter and for people with Black or Asian ethnic backgrounds.

For peer review only

For peer review only - http://bmjopen.bmj.com/site/about/guidelines.xhtml

Tables

Table 1 Comparison of included and excluded participants

Characteristics	Included (N=449,943)	Excluded (N=52,550)
Sex		
Female	240,976 (53.6%)	32,402 (61.7%)
Male	208,967 (46.4%)	20,148 (38.3%)
Average age (SD)	56.5 (8.1)	57.0 (7.8)
BMI[1]		
Underweight	2,307 (0.5%)	321 (0.6%)
Healthy weight	145,082 (32.3%)	15,222 (29.7%)
Overweight	190,177 (42.4%)	22,053 (43.1%)
Obese	110,541 (24.7%)	13,600 (26.6%)
Central obesity[1]		
Not obese	177,869 (39.6%)	17,719 (34.5%)
Central obesity	271,126 (60.4%)	33,642 (65.5%)
Ethnic background		

For peer review only

For peer review only - http://bmjopen.bmj.com/site/about/guidelines.xhtml

White	424,213 (94.4%)	47,901 (91.3%)
Mixed	2,593 (0.6%)	316 (0.6%)
Asian	8,016 (1.8%)	1,823 (3.5%)
Black	7,050 (1.6%)	984 (1.9%)
Chinese	1,407 (0.3%)	167 (0.3%)
Other ethnicity[2]	6,068 (1.4%)	1,267 (2.4%)
Tobacco smoking		
Non-smoker	402,337 (89.6%)	45,860 (88.3%)
Current smoker	46,793 (10.4%)	6,184 (11.9%)
Alcohol drinking status		
Never drink	35,590 (7.9%)	5,071 (9.8%)
Drink occasionally	101,690 (22.7%)	12,208 (23.5%)
Drink weekly	220,709 (49.2%)	24,028 (46.2%)
Drink daily	90,984 (20.3%)	10,713 (20.6%)
Vitamin and mineral supplementation use		

For peer review only - http://bmjopen.bmj.com/site/about/guidelines.xhtml

Vitamin D and associated mineral supplement[3]	285,524 (63.6%)	31,097 (63.9%)
Other vitamin and mineral supplement[4]	163,214 (36.4%)	17,537 (36.1%)
Mean outdoors time in summer (SD)	3.8 (2.4)	3.8 (2.4)
Mean outdoors time in winter (SD)	1.9 (1.8)	1.9 (1.8)
Index of multiple deprivation index (IMD)[5]		
1 (Least deprived)	88,516 (20.2%)	9,616 (18.7%)
2	88,210 (20.1%)	9,782 (19.1%)
3	87,584 (20.0%)	10,249 (20.0%)
4	87,585 (20%)	10,306 (21.0%)
5 (Most deprived)	86,539 (19.7%)	11,370 (22.2%)

1. The classification is suggested by NICE guidelines; 2. Includes any other unlisted ethnic groups, unclear ethnic groups and participants prefer not to answer; 3. Vitamin D and associated mineral: vitamin D, multivitamin, fish oil, and calcium supplementation; 4. Other vitamin and mineral supplements: vitamin A, B, C, E, folic acid or folate, glucosamine, zinc, iron, selenium and other supplements; 5. IMD scores were by quintile.

For peer review only

For peer review only - http://bmjopen.bmj.com/site/about/guidelines.xhtml

Table 2 Basic characteristics of the study population

Characteristics	Vitamin D Deficiency (< 25 nmol/L)	Vitamin D Insufficiency (25 – 50 nmol/L)	Vitamin D Sufficiency (> 50 nmol/L)
Serum vitamin D levels (SD) (nmol/L)	19.2 (3.9)	37.8 (7.1)	67.7 (14.5)
Sex			
Female (N=240,263)	32,180 (13.4%)	100,423 (41.8%)	107,660 (44.8%)
Male (N=208,338)	28,507 (13.7%)	87,368 (41.9%)	92,463 (44.4%)
Age (SD)	54.8 (8.2)	56.2 (8.1)	57.3 (8.0)
BMI[1]			
Underweight (N=2,301)	423 (18.3%)	844 (36.7%)	1,034 (45%)
Healthy weight (N=144,591)	15,886 (11.0%)	54,115 (37.4%)	74,590 (51.6%)
Overweight (N=189,583)	22,841 (12.1%)	79,351 (41.9%)	87,391 (46.1%)
Obese (N=110,292)	20,990 (19.0%)	52,704 (47.8%)	36,598 (33.2%)
Central obesity[1]			

For peer review only - http://bmjopen.bmj.com/site/about/guidelines.xhtml

Non-obese (N=177,348)	19,514 (11.0%)	66,998 (37.8%)	90,836 (51.2%)
Central obesity (N=270,306)	40,903 (15.1%)	120,389 (44.5%)	109,014 (40.3%)
Ethnic background			
White (N=422,907)	50,920 (12.0%)	176,195 (41.7%)	195,792 (46.3%)
Mixed (N=2,589)	642 (24.8%)	1,241 (47.9%)	706 (27.3%)
Asian (N=8,000)	4,297 (53.7%)	2,979 (37.3 %)	724 (9.1%)
Black (N=7,046)	2,459 (34.9%)	3,503 (49.7%)	1,084 (15.4%)
Chinese (N=1,405)	381 (27.1%)	787 (56.0%)	237 (16.9%)
Other ethnicity[2] (N=6,059)	1,860 (30.7%)	2,816 (46.5%)	1,383 (22.8%)
Tobacco smoking			
Non-smoker (N=401,069)	50,270 (12.5%)	167,496 (41.8%)	183,303 (45.7%)
Current smoker (N=46,721)	10,170 (21.7%)	19,944 (42.7%)	16,607 (35.6%)
Alcohol drinking status			
Never drink (N=35,503)	8,419 (23.7%)	15,265 (43.0%)	11,819 (33.3%)
Drink occasionally (N=101,398)	16,655 (16.4%)	45,208 (44.6%)	39,535 (39.0%)
Drink weekly (N=219,988)	24,577 (11.1%)	90,836 (41.3%)	104,575 (47.6%)

For peer review only - http://bmjopen.bmj.com/site/about/guidelines.xhtml

Drink daily (N=90,742)	10,730 (11.8%)	36,063 (39.7%)	43,949 (48.4%)
Mean outdoors time in summer (hours) (SD)	3.3 (2.4)	3.6 (2.4)	4.1 (2.4)
Mean outdoors time in winter (hours) (SD)	1.7 (1.8)	1.9 (1.8)	2.1 (1.8)
Vitamin and mineral supplementation use			
Other vitamin or mineral supplement[3] (N=284,768)	48,877 (17.1%)	128,478 (45.1%)	107,413 (37.7%)
Vitamin D and associated mineral[4] supplement (N=162,629)	11,368 (7.0%)	58,801 (36.2%)	92,460 (56.9%)
Index of multiple deprivation (IMD)[5]			
1 (Least deprived, N=88,264)	8,414 (9.5%)	34,962 (39.6%)	44,888 (50.9%)
2 (N=87,909)	9,321 (10.6%)	36,073 (41.0%)	42,515 (48.4%)
3 (N=87,268)	10,480 (12.0%)	36,049 (41.3%)	40,739 (46.7%)
4 (N=87,321)	13,211 (15.1%)	37,646 (43.1%)	36,464 (41.8%)
5 (Most deprived N=86,347)	17,602 (20.4%)	38,369 (44.4%)	30,376 (35.2%)
Seasons			
Spring (N= 129,570)	25,912 (20%)	62,378 (48.1%)	41,280 (31.7%)

For peer review only - http://bmjopen.bmj.com/site/about/guidelines.xhtml

Summer (N= 118,924)	5,385 (4.5%)	39,856 (33.5%)	73,683 (62.0%)
Autumn (N= 108,888)	8,324 (7.6%)	41,496 (38.1%)	59,068 (54.3%)
Winter (N= 91,219)	21,066 (23.1%)	44,061 (48.3%)	26,092 (28.6%)
Regions of UK Biobank assessment centres			
South West (N= 38,872)	3,068 (7.9%)	14,622 (37.6%)	21,182 (54.5%)
South East (N= 39,814)	3,245 (8.2%)	15,400 (38.7%)	21,169 (53.2%)
London (N= 61,291)	10,232 (16.7%)	27,037 (44.1%)	24,022 (39.2%)
East Midlands (N= 30,337)	3,001 (9.9%)	12,115 (39.9%)	15,221 (50.2%)
West Midlands (N= 40,044)	6,785 (17.0%)	17,670 (44.1%)	15,589 (38.9%)
Wales (N= 19,142)	2,732 (14.3%)	8,808 (46.0%)	7,614 (39.8%)
Yorkshire and The Humber (N= 66,197)	8,372 (12.7%)	27,878 (42.1%)	29,947 (45.2%)
North West (N= 68,196)	8,715 (12.8%)	27,924 (41.0%)	31,557 (46.3%)
North East (N=52,277)	6,919 (13.2%)	21,174(40.5%)	24,184 (46.3%)
Scotland (N=32,419)	7,618 (23.5%)	15,163 (46.8%)	9,638 (29.7%)

1. The classification is suggested by NICE guidelines; 2. Includes any other unlisted ethnic groups, unclear ethnic groups and participants prefer not to answer; 3. Vitamin D and associated mineral: vitamin D, multivitamin, fish oil, and calcium supplementation; 4. Other vitamin and mineral supplements: vitamin A, B, C, E, folic acid or folate, glucosamine, zinc, iron, selenium and other supplements; 5. IMD scores were by quintile.

For peer review only - http://bmjopen.bmj.com/site/about/guidelines.xhtml

Table 3 The association between demographic characteristics and low vitamin D status

Characteristics	Odds ratio of vitamin D insufficiency (25(OH)D < 50 nmol/L)		Odds ratio of vitamin D deficiency (25(OH)D < 25 nmol/L)	
	Crude	Adjusted[1]	Crude	Adjusted[1]
Sex				
Female	1	1	1	1
Male	1.02 (1.0, 1.03)	0.91 (0.90, 0.93)	1.02 (1.01, 1.04)	0.91 (0.9, 0.93)
Age (SD)	0.98 (0.97, 0.97)	0.98 (0.98, 0.98)	0.97 (0.96, 0.97)	0.98 (0.98, 0.98)
BMI				
Healthy weight	1	1	1	1
Underweight	1.3 (1.20, 1.42)	1.26 (1.14, 1.38)	1.83 (1.64, 2.03)	1.71 (1.51, 1.93)
Overweight	1.25 (1.23, 1.26)	1.24 (1.22, 1.25)	1.11 (1.09, 1.13)	1.04 (1.01, 1.06)
Obese	2.15 (2.11, 2.18)	2.08 (2.04, 2.12)	1.91 (1.86, 1.95)	1.68 (1.64, 1.72)
Ethnic background				
White	1	1	1	1
Mixed	2.30 (2.11, 2.51)	2.24 (2.03, 2.46)	2.42 (2.21. 2.64)	2.31 (2.09, 2.56)

For peer review only - http://bmjopen.bmj.com/site/about/guidelines.xhtml

For peer review only

Asian	8.67 (8.02, 9.35)	8.54 (7.87, 9.27)	8.47 (8.10,8.86)	10.99 (10.39, 11.62)
Black	4.74 (4.44, 5.06)	4.14 (3.85, 4.45)	3.93 (3.74, 4.13)	3.6 (3.39, 3.83)
Chinese	4.25 (3.70, 4.89)	4.42 (3.81, 5.14)	2.72 (2.42, 3.06)	2.77 (2.42, 3.18)
Other ethnicity	2.91 (2.74, 3.10)	2.73 (2.55, 2.93)	3.24 (3.07, 3.42)	3.11 (2.9, 3.33)
Tobacco smoking				
Non-smoker	1	1	1	1
Current smoker	1.53 (1.50, 1.56)	1.43 (1.40, 1.46)	1.94 (1.90, 1.99)	1.82 (1.77, 1.87)
Alcohol drinking status				
Never drink	1	1	1	1
Drink occasionally	0.78 (0.76, 0.8)	0.85 (0.84, 0.88)	0.63 (0.61, 0.65)	0.75 (0.73, 0.78)
Drink weekly	0.55 (0.53, 0.56)	0.66 (0.64, 0.68)	0.40 (0.39, 0.42)	0.55 (0.53, 0.57)
Drink daily	0.53 (0.52, 0.55)	0.70 (0.68, 0.72)	0.43 (0.42, 0.45)	0.66 (0.64, 0.69)
Vitamin and mineral supplementation				
Other vitamin and mineral supplement	1	1	1	1

For peer review only - http://bmjopen.bmj.com/site/about/guidelines.xhtml

Vitamin D, multivitamin, fish oil and calcium supplement	0.46 (0.45, 0.47)	0.41 (0.41, 0.42)	0.36 (0.35, 0.37)	0.32 (0.31, 0.33)
Index of multiple deprivation (IMD)				
1 (Least deprived)	1	1	1	1
2	1.11 (1.09, 1.13)	1.03 (1.01, 1.05)	1.12 (1.09,1.16)	1.02 (0.98, 1.05)
3	1.18 (1.16, 1.2)	1.05 (1.03,1.07)	1.29 (1.26,1.33)	1.11 (1.07, 1.14)
4	1.44 (1.42, 1.47)	1.17 (1.14,1.19)	1.69 (1.64,1.74)	1.27 (1.23, 1.31)
5 (Most deprived)	1.91 (1.87, 1.94)	1.34 (1.31,1.37)	2.43 (2.36, 2.50)	1.53 (1.48, 1.58)
Seasons				
Summer	1	1	1	1
Spring	3.48 (3.42, 3.54)	3.86 (3.79, 3.93)	5.26 (5.1, 5.42)	6.43 (6.22, 6.65)
Autumn	1.37 (1.35, 1.40)	1.43 (1.40, 1.45)	1.74 (1.68, 1.80)	1.89 (1.82, 1.96)
Winter	4.06 (3.99, 4.14)	4.56 (4.47, 4.65)	6.31 (6.12, 6.51)	7.82 (7.55, 8.1)
Regions (categorized centres)				
South West	1	1	1	1
South East	1.05 (1.03, 1.08)	1.15 (1.11, 1.18)	1.04 (0.98, 1.09)	1.11 (1.05, 1.18)

For peer review only

London	1.86 (1.81, 1.91)	1.31 (1.28, 1.35)	2.34 (2.24, 2.44)	1.31 (1.25, 1.38)
East Midlands	1.19 (1.15, 1.23)	1.07 (1.04, 1.11)	1.28 (1.22, 1.35)	1.13 (1.07, 1.2)
West Midlands	1.88 (1.82, 1.93)	1.22 (1.18, 1.26)	2.38 (2.27, 2.48)	1.26 (1.19, 1.32)
Wales	1.81 (1.75, 1.88)	1.03 (0.99, 1.07)	1.94 (1.84, 2.05)	1.03 (0.97, 1.09)
Yorkshire and The Humber	1.45 (1.41, 1.48)	1.19(1.16, 1.23)	1.68 (1.61, 1.76)	1.30 (1.24, 1.36)
North West	1.38 (1.35, 1.42)	1.15 (1.12, 1.18)	1.68 (1.61, 1.76)	1.28 (1.22, 1.34)
North East	1.39 (1.35, 1.43)	1.23 (1.19, 1.27)	1.78 (1.7, 1.86)	1.53 (1.46, 1.61)
Scotland	2.83 (2.74, 2.92)	1.98 (1.91, 2.05)	3.58 (3.43, 3.75)	2.39 (2.28, 2.51)

1. Adjusted for sex, age, BMI categories, ethnicity, smoking, drinking, vitamin D supplementation, index of multiple deprivation, seasons, regions of UK Biobank assessment centres; 3. IMD scores were by quintile.

For peer review only

For peer review only - http://bmjopen.bmj.com/site/about/guidelines.xhtml

326 Authors' contribution

327 LYL contributed to the design of the study, analysed the data, drafted and revised the
328 manuscript according to other authors' comments; CWG contributed to the design of the
329 study, made critical comments on the manuscript and revised the paper critically; SML
330 contributed to the design of the study, made critical comments on the manuscript and
331 revised the paper critically; LS contributed to the design of the study and revised the paper
332 critically. All authors approved the final version of the protocol.

333 Competing interest:

334 None declared.

335 Funding statement:

336 LYL is funded by the scholarship of government sponsorship for overseas study by the
337 Ministry of Education Republic of China (Taiwan). CWG is supported by a Wellcome
338 Intermediate Clinical Fellowship (201440_Z_16_Z). SML is funded by a Wellcome Senior
339 Clinical Fellowship in Science (205039/Z/16/Z).

340 Data sharing statement:

341 Other researchers can apply for UK Biobank data to answer specific research questions.
342 We will upload our analysis codes to LSHTM Data Compass, an open data repository for
343 research outputs from LSHTM (https://datacompass.lshtm.ac.uk/).

344 Acknowledgement

345 We thank Yi-Tin Ching for assistance with technical support of ArcGIS mapping.

346 Patient consent for publication:

347 Not required

For peer review only

References:

1. Holick MF. Vitamin D deficiency. *The New England journal of medicine* 2007;357(3):266-81. doi: 10.1056/NEJMra070553 [published Online First: 2007/07/20]
2. Public Health England. PHE publishes new advice on vitamin D 2016 [Available from: https://www.gov.uk/government/news/phe-publishes-new-advice-on-vitamin-d accessed 05 October 2020.
3. Sassi F, Tamone C, D'Amelio P. Vitamin D: Nutrient, Hormone, and Immunomodulator. *Nutrients* 2018;10(11) doi: 10.3390/nu10111656 [published Online First: 2018/11/08]
4. Mann EH, Pfeffer PE, Hawrylowicz CM. Chapter 104 - Vitamin D and Adaptive Immunology in Health and Disease. In: Feldman D, ed. Vitamin D (Fourth Edition): Academic Press 2018:937-49.
5. Martineau AR, Jolliffe DA, Hooper RL, et al. Vitamin D supplementation to prevent acute respiratory tract infections: systematic review and meta-analysis of individual participant data. *Bmj* 2017;356:i6583. doi: https://dx.doi.org/10.1136/bmj.i6583
6. The Scientific Advisory Committee on Nutrition. Vitamin D and Health: Public Health England 2016.
7. Royal Osteoporosis Society. Vitamin D and Bone Health: A Practical Clinical Guideline for Patient Management. 2 ed: Royal Osteoporosis Society,, 2018:10.
8. Hypponen E, Power C. Hypovitaminosis D in British adults at age 45 y: nationwide cohort study of dietary and lifestyle predictors. *The American journal of clinical nutrition* 2007;85(3):860-8. doi: 10.1093/ajcn/85.3.860 [published Online First: 2007/03/09]
9. Public Health England. National Diet and Nutrition Survey Results from Years 1, 2, 3 and 4 (combined) of the Rolling Programme (2008/2009 – 2011/2012). In: Bates B, Lennox A, Prentice A, et al., eds. National Diet and Nutrition Survey (NDNS): Public Health England,, 2017.
10. Sudlow C, Gallacher J, Allen N, et al. UK biobank: an open access resource for identifying the causes of a wide range of complex diseases of middle and old age. *PLoS Med* 2015;12(3):e1001779. doi: 10.1371/journal.pmed.1001779 [published Online First: 2015/04/01]
11. UK Biobank. UK Biobank Biomarker Panel, 2015.
12. Fry A, Littlejohns TJ, Sudlow C, et al. Comparison of Sociodemographic and Health-Related Characteristics of UK Biobank Participants With Those of the General Population. *American journal of epidemiology* 2017;186(9):1026-34. doi: https://dx.doi.org/10.1093/aje/kwx246
13. UK Biobank. Index of Multiple Deprivation Scores 2018.
14. Elliott P, Peakman TC, Biobank UK. The UK Biobank sample handling and storage protocol for the collection, processing and archiving of human blood and urine. *International journal of epidemiology* 2008;37(2):234-44. doi: 10.1093/ije/dym276 [published Online First: 2008/04/03]
15. CDC. CDC Vitamin D Standardization-Certification Program (CDC VDSCP), 2020.
16. UK Biobank. Biomarker assay quality procedures: approaches used to minimise systematic and random errors. 1.2 ed, 2019.
17. UK Biobank. Companion document for serum biomarker data. 1 ed, 2019.
18. Pearce SH, Cheetham TD. Diagnosis and management of vitamin D deficiency. *Bmj* 2010;340:b5664. doi: 10.1136/bmj.b5664
19. National Institute for Health and Care Excellence (NICE). BMI: preventing ill health and premature death in black, Asian and other minority ethnic groups: National Institute for Health and Clinical Excellence 2013.
20. Office for National Statistics. Regions (December 2017) Map in United Kingdom 2019 [Available from: https://geoportal.statistics.gov.uk/datasets/0758ee9479044ecc9547ebab340c84a8 accessed 09/23 2020.
21. Met Office. Seasons 2019 [Available from: https://www.metoffice.gov.uk/weather/learn-about/weather/seasons accessed 25/11 2019.
22. World Medical A. World Medical Association Declaration of Helsinki: ethical principles for medical research involving human subjects. *JAMA* 2013;310(20):2191-94. doi: 10.1001/jama.2013.281053
23. Darling AL, Hart KH, Macdonald HM, et al. Vitamin D deficiency in UK South Asian Women of childbearing age: a comparative longitudinal investigation with UK Caucasian women. *Osteoporos Int* 2013;24(2):477-88. doi: 10.1007/s00198-012-1973-2 [published Online First: 2012/04/25]

For peer review only

24. Renzaho AMN, Halliday JA, Nowson C. Vitamin D, obesity, and obesity-related chronic disease among ethnic minorities: A systematic review. *Nutrition* 2011;27(9):868-79. doi: https://doi.org/10.1016/j.nut.2010.12.014
25. Santos A, Amaral TF, Guerra RS, et al. Vitamin D status and associated factors among Portuguese older adults: results from the Nutrition UP 65 cross-sectional study. *BMJ open* 2017;7(6):e016123. doi: 10.1136/bmjopen-2017-016123 [published Online First: 2017/06/25]
26. Liu X, Baylin A, Levy PD. Vitamin D deficiency and insufficiency among US adults: prevalence, predictors and clinical implications. *Br J Nutr* 2018;119(8):928-36. doi: 10.1017/s0007114518000491 [published Online First: 2018/04/13]
27. Aspell N, Laird E, Healy M, et al. The Prevalence and Determinants of Vitamin D Status in Community-Dwelling Older Adults: Results from the English Longitudinal Study of Ageing (ELSA). *Nutrients* 2019;11(6):1253. doi: 10.3390/nu11061253
28. Tardelli VS, Lago M, Silveira DXD, et al. Vitamin D and alcohol: A review of the current literature. *Psychiatry Res* 2017;248:83-86. doi: 10.1016/j.psychres.2016.10.051 [published Online First: 2016/12/30]

For peer review only

416 # Supplementary figures

417 Supplementary figure 1 Mean percentage of participants with vitamin D deficiency by UK
418 Biobank assessment centres and season

For peer review only

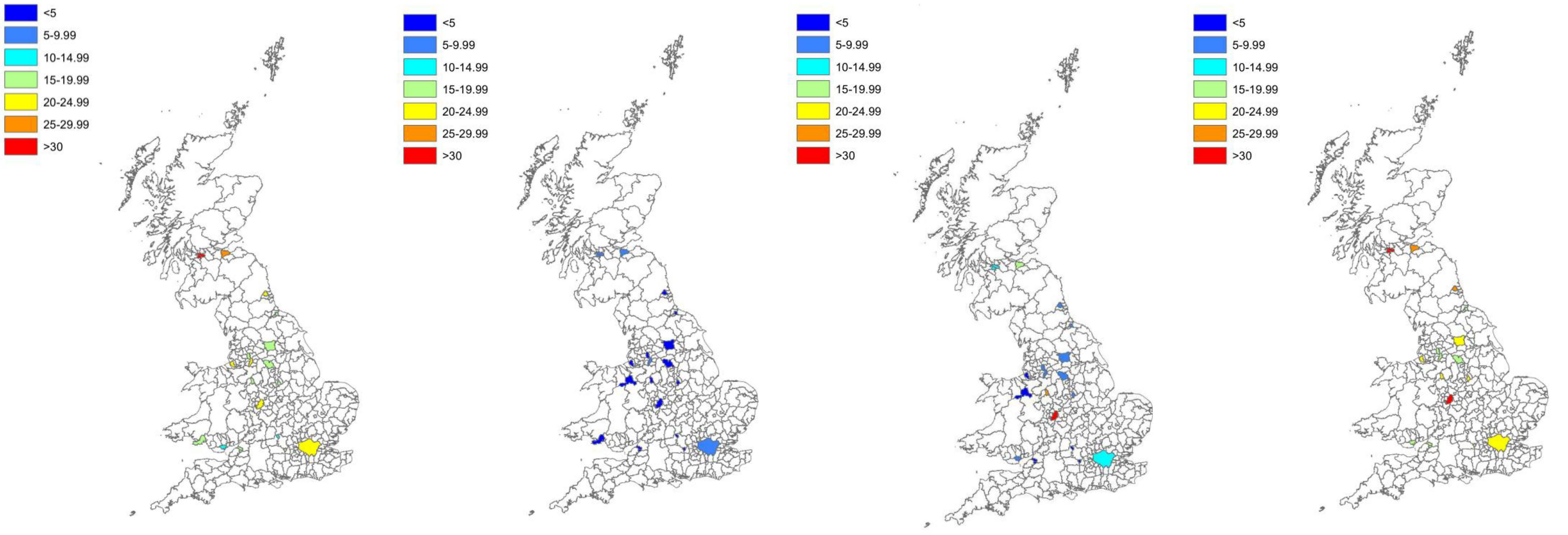

a. Spring b. Summer c. Autumn d. Winter

STROBE 2007 (v4) checklist of items to be included in reports of observational studies in epidemiology*
Checklist for cohort, case-control, and cross-sectional studies (combined)

Section/Topic	Item #	Recommendation	Reported on page #
**Title and abstract**	1	(*a*) Indicate the study's design with a commonly used term in the title or the abstract	#1
		(*b*) Provide in the abstract an informative and balanced summary of what was done and what was found	#4
**Introduction**			
Background/rationale	2	Explain the scientific background and rationale for the investigation being reported	#6
Objectives	3	State specific objectives, including any pre-specified hypotheses	#6
**Methods**			
Study design	4	Present key elements of study design early in the paper	#8
Setting	5	Describe the setting, locations, and relevant dates, including periods of recruitment, exposure, follow-up, and data collection	#7
Participants	6	(*a*) *Cohort study*—Give the eligibility criteria, and the sources and methods of selection of participants. Describe methods of follow-up<br>*Case-control study*—Give the eligibility criteria, and the sources and methods of case ascertainment and control selection. Give the rationale for the choice of cases and controls<br>*Cross-sectional study*—Give the eligibility criteria, and the sources and methods of selection of participants	#7
		(*b*) *Cohort study*—For matched studies, give matching criteria and number of exposed and unexposed<br>*Case-control study*—For matched studies, give matching criteria and the number of controls per case	Not applicable
Variables	7	Clearly define all outcomes, exposures, predictors, potential confounders, and effect modifiers. Give diagnostic criteria, if applicable	#7
Data sources/ measurement	8*	For each variable of interest, give sources of data and details of methods of assessment (measurement). Describe comparability of assessment methods if there is more than one group	#7
Bias	9	Describe any efforts to address potential sources of bias	#8
Study size	10	Explain how the study size was arrived at	#7

For peer review only

Quantitative variables	11	Explain how quantitative variables were handled in the analyses. If applicable, describe which groupings were chosen and why	#7
Statistical methods	12	(*a*) Describe all statistical methods, including those used to control for confounding	#8
		(*b*) Describe any methods used to examine subgroups and interactions	Not applicable
		(*c*) Explain how missing data were addressed	Not applicable
		(*d*) *Cohort study*—If applicable, explain how loss to follow-up was addressed<br>*Case-control study*—If applicable, explain how matching of cases and controls was addressed<br>*Cross-sectional study*—If applicable, describe analytical methods taking account of sampling strategy	Not applicable
		(*e*) Describe any sensitivity analyses	Not applicable
**Results**			
Participants	13*	(a) Report numbers of individuals at each stage of study—eg numbers potentially eligible, examined for eligibility, confirmed eligible, included in the study, completing follow-up, and analysed	#9
		(b) Give reasons for non-participation at each stage	#9
		(c) Consider use of a flow diagram	
Descriptive data	14*	(a) Give characteristics of study participants (eg demographic, clinical, social) and information on exposures and potential confounders	#10
		(b) Indicate number of participants with missing data for each variable of interest	#13
		(c) *Cohort study*—Summarise follow-up time (eg, average and total amount)	Not applicable
Outcome data	15*	*Cohort study*—Report numbers of outcome events or summary measures over time	Not applicable
		Case-control study—Report numbers in each exposure category, or summary measures of exposure	Not applicable
		Cross-sectional study—Report numbers of outcome events or summary measures	#10, #15
Main results	16	(*a*) Give unadjusted estimates and, if applicable, confounder-adjusted estimates and their precision (eg, 95% confidence interval). Make clear which confounders were adjusted for and why they were included	Not applicable
		(*b*) Report category boundaries when continuous variables were categorized	#15

For peer review only

		(*c*) If relevant, consider translating estimates of relative risk into absolute risk for a meaningful time period	Not applicable
Other analyses	17	Report other analyses done—eg analyses of subgroups and interactions, and sensitivity analyses	Not applicable
**Discussion**			
Key results	18	Summarise key results with reference to study objectives	#10
Limitations	19	Discuss limitations of the study, taking into account sources of potential bias or imprecision. Discuss both direction and magnitude of any potential bias	#11
Interpretation	20	Give a cautious overall interpretation of results considering objectives, limitations, multiplicity of analyses, results from similar studies, and other relevant evidence	#11
Generalisability	21	Discuss the generalisability (external validity) of the study results	#12
**Other information**			
Funding	22	Give the source of funding and the role of the funders for the present study and, if applicable, for the original study on which the present article is based	#2

*Give information separately for cases and controls in case-control studies and, if applicable, for exposed and unexposed groups in cohort and cross-sectional studies.
Note: An Explanation and Elaboration article discusses each checklist item and gives methodological background and published examples of transparent reporting. The STROBE checklist is best used in conjunction with this article (freely available on the Web sites of PLoS Medicine at http://www.plosmedicine.org/, Annals of Internal Medicine at http://www.annals.org/, and Epidemiology at http://www.epidem.com/). Information on the STROBE Initiative is available at www.strobe-statement.org.

For peer review only
